# A Relativistic Theory of Consciousness

**DOI:** 10.3389/fpsyg.2021.704270

**Published:** 2022-05-12

**Authors:** Nir Lahav, Zachariah A. Neemeh

**Affiliations:** ^1^Department of Physics, Bar-Ilan University, Ramat Gan, Israel; ^2^Department of Philosophy, The University of Memphis, Memphis, TN, United States; ^3^Institute for Intelligent Systems, The University of Memphis, Memphis, TN, United States

**Keywords:** consciousness, phenomenology, qualia, relativity (physics), the hard problem of consciousness, mathematical formulization of consciousness

## Abstract

In recent decades, the scientific study of consciousness has significantly increased our understanding of this elusive phenomenon. Yet, despite critical development in our understanding of the functional side of consciousness, we still lack a fundamental theory regarding its phenomenal aspect. There is an “explanatory gap” between our scientific knowledge of functional consciousness and its “subjective,” phenomenal aspects, referred to as the “hard problem” of consciousness. The phenomenal aspect of consciousness is the first-person answer to “what it’s like” question, and it has thus far proved recalcitrant to direct scientific investigation. Naturalistic dualists argue that it is composed of a primitive, private, non-reductive element of reality that is independent from the functional and physical aspects of consciousness. Illusionists, on the other hand, argue that it is merely a cognitive illusion, and that all that exists are ultimately physical, non-phenomenal properties. We contend that both the dualist and illusionist positions are flawed because they tacitly assume consciousness to be an absolute property that doesn’t depend on the observer. We develop a conceptual and a mathematical argument for a relativistic theory of consciousness in which a system either has or doesn’t have phenomenal consciousness *with respect to some observer.* Phenomenal consciousness is neither private nor delusional, just relativistic. In the frame of reference of the cognitive system, it will be observable (first-person perspective) and in other frame of reference it will not (third-person perspective). These two cognitive frames of reference are both correct, just as in the case of an observer that claims to be at rest while another will claim that the observer has constant velocity. Given that consciousness is a relativistic phenomenon, neither observer position can be privileged, as they both describe the same underlying reality. Based on relativistic phenomena in physics we developed a mathematical formalization for consciousness which bridges the explanatory gap and dissolves the hard problem. Given that the first-person cognitive frame of reference also offers legitimate observations on consciousness, we conclude by arguing that philosophers can usefully contribute to the science of consciousness by collaborating with neuroscientists to explore the neural basis of phenomenal structures.

## Introduction

### The Hard Problem of Consciousness

As one of the most complex structures we know of nature, the brain poses a great challenge to us in understanding how higher functions like perception, cognition, and the self arise from it. One of its most baffling abilities is its capacity for conscious experience (van Gulick, 2014). Thomas Nagel (1974) suggests a now widely accepted definition of consciousness: a being is conscious just if there is “something that it is like” to be that creature, i.e., some subjective way the world seems or appears from the creature’s point of view. For example, if bats are conscious, that means there is something it is like for a bat to experience its world through its echolocational senses. On the other hand, under deep sleep (with no dreams) humans are unconscious because there is nothing it is like for humans to experience their world in that state.

In the last several decades, consciousness has transformed from an elusive metaphysical problem into an empirical research topic. Nevertheless, it remains a puzzling and thorny issue for science. At the heart of the problem lies the question of the brute phenomena that we experience from a first-person perspective—e.g., what it is like to feel redness, happiness, or a thought. These qualitative states, or qualia, compose much of the phenomenal side of consciousness. These qualia are arranged into spatial and temporal patterns and formal structures in phenomenal experience, called eidetic or transcendental structures^1^. For example, while qualia pick out how a specific note sounds, eidetic structures refer to the temporal form of the whole melody. Hence, our inventory of the elusive properties of phenomenal consciousness includes both qualia and eidetic structures.

One of the central aspects of phenomenal features is privacy. It seems that my first-person feeling of happiness, for example, cannot be measured from any other third-person perspective. One can take indirect measurements of my heart rate or even measure the activity in the brain networks that create the representation of happiness in my mind, but these are just markers of the feeling that I have, and not the feeling itself (Block, 1995). From my first-person perspective, I don’t feel the representation of happiness. I just feel happiness. Philosophers refer to this feature of phenomenal consciousness as “transparency”: we seem to directly perceive things, rather than mental representations, even though mental representations mediate experience. But this feeling cannot be directly measured from the third-person perspective, and so is excluded from scientific inquiry. Yet, let us say that we identify a certain mental representation in a subject’s brain as “happiness.” What justifies us in calling it “happiness” as opposed to “sadness?” Perhaps it is correlated with certain physiological and behavioral measures. But, ultimately, the buck stops somewhere: at a subject’s phenomenological report otherwise, we cannot know what this representation represents (Gallagher and Zahavi, 2020). If we categorize the representation as “happiness” but the subject insists they are sad, we are probably mistaken, not the subject. Phenomenal properties are seemingly private and, by some accounts, even beyond any physical explanation. For being physical means being public and measurable and is epistemically inconsistent with privacy. Take an electron, for example. We know it is physical because we can measure it with e.g., a cathode ray tube. If electrons were something that only I could perceive (i.e., if they were private), then we would not include them as physical parts of the scientific worldview. Affective scientists, for example, measure aspects of feeling all the time, like valence and arousal, but measuring these is not the same as measuring the feeling of happiness itself. What affective scientists measure are outcomes of the feeling, while the feeling itself is private and not measurable by the scientists. To date, it is not clear how to bridge the “explanatory gap” (Levine, 1983) between “private” phenomenal features and public, measurable features (e.g., neurocomputational structures), leaving us stuck in the “hard problem” of consciousness (Chalmers, 1996). This gap casts a shadow on the possibility for neuroscience to solve the hard problem because all explanations will always remain within a third-person perspective (e.g., neuronal firing patterns and representations), leaving the first-person perspective out of the reach of neuroscience. This situation divides consciousness into two separate aspects, the functional aspect and the phenomenal one (Block, 1995). The functional aspect (‘functional consciousness’), is the objectively observable aspect of consciousness (Franklin et al., 2016; Kanai et al., 2019). [In that sense, it’s similar to Ned Block’s definition of access conscious, but with less constraints. All phenomenal consciousness has a functional aspect and vice versa, whereas for Block this strict equivalence doesn’t hold (Block, 2011).] But the subjective aspect (phenomenal consciousness) is not directly observable except on the part of the person experiencing that conscious state. As we saw above, because they are private, phenomenal properties are distinct from any cognitive and functional property (which can be publicly observable). Any theory of consciousness should explain how to bridge this gap—How can functional, public, aspects give rise to phenomenal, private aspects?

Nevertheless, in recent decades, consciousness has become increasingly amenable to empirical investigation by focusing on its functional aspect, finally enabling us to begin to understand this enigmatic phenomenon. For example, we now have good evidence that consciousness doesn’t occur in a single brain area. Rather, it seems to be a global phenomenon in widespread areas of the brain (Baars, 1988; Varela et al., 2001; Mashour et al., 2020).

In studying *functional consciousness*, we take consciousness to be a form of information processing and manipulation of representations, and we trace its functional or causal role within the cognitive system. Widely successful theories, such as global workspace theory (Baars, 1988; Dehaene, 2014; Franklin et al., 2016), attention schema theory (Graziano, 2019), recurrent processing theory (Lamme, 2006; Fahrenfort et al., 2007), and integrated information theory (Tononi, 2008; Oizumi et al., 2014) are virtually premised on this information processing account. Despite our advances in the study of functional consciousness, we still lack a convincing way to bridge the explanatory gap to phenomenal consciousness. The questions, “Why does it feel like anything at all to process information?”, and “How can this feeling be private?” still remain controversial. The hard problem primarily remains a philosophical rather than scientific question. Phenomenal consciousness must be the ultimate reference point for any scientific theory of consciousness. Ultimately, theories of consciousness as information processing, i.e., theories of functional consciousness, only approximate full-blown consciousness by abstracting away its phenomenal features. But they must ultimately refer to phenomenal features in order to give a full explanation of consciousness. Otherwise, there are no grounds for labeling them theories of consciousness, rather than theories of cognition or global informational access.

Let’s take Integrated Information Theory (IIT), for example. IIT claims to bridge between the phenomenal and functional aspects of consciousness and to answer the question of what qualia are (Tononi et al., 2016). According to IIT, consciousness is the result of highly integrated information in the brain [which is “the amount of information generated by a complex of elements, above and beyond the information generated by its parts” (Tononi, 2008)]. A mathematical formula can determine how much integrated information, and thus how much consciousness, is present in any specific system. This measure is referred to as *ϕ*, and if *ϕ* is higher than zero, the system is conscious. In this situation, the system has a “maximally irreducible conceptual structure” which is identical to its phenomenal experience (Oizumi et al., 2014). These maximally irreducible conceptual structures are composed of integrated information states, and thus a quale is identical to a specific relation between integrated information states. The problem is that these maximally irreducible conceptual structures are physical states and even though they can be very complex, they are still public and observable from a third-person perspective, while qualia are private and only perceptible from the first-person perspective. How can there be an identity between opposite properties like public and privacy properties? Yet, this is what IIT suggests without any explanation about how to bridge this contradiction. Let’s choose a conceptual structure such that it is a quale of happiness according to IIT. However, it cannot truly be the quale of happiness because, in principle, when we measure it, we observe a physical process and not the qualitative happiness itself (that’s why it’s not enough to measure the physical process, and eventually we need to ask the participants what they feel). Again, we remain stuck within the explanatory gap and the hard problem. In other words, IIT hasn’t solved the hard problem and hasn’t bridged the explanatory gap between private and public properties (Mindt, 2017). In order to avoid this problem, IIT needs to assume phenomenal consciousness as a primitive element separated from other physical elements like space and time. In that case, there is no gap to bridge because now these maximally irreducible conceptual structures are not qualia anymore. They are just physical structures that have properties that correspond to the properties of phenomenal consciousness, but that are not identical to them. In that sense, IIT just shows correspondence between two separate kinds of elements, phenomenal and physical. In the next paragraph we will see that this kind of solution is exactly what Chalmers refers to as ‘naturalistic dualism.’ The problem with that is that instead of understanding what phenomenal consciousness is and what the relations are between the physical world and the phenomenal world, it just assumes that phenomenal consciousness exists as a separate basic element in the world, and it does not solve how there can be any interaction between physical and phenomenal elements (Carroll, 2021).

This is not an issue of only IIT, but a problem to all physicalist’s theories. As David Chalmers (1995) has put it, “the structure and dynamics of physical processes yield only more structure and dynamics, so structures and functions are all we can expect these processes to explain.” The structure and dynamics of information tell us nothing of the story of how one gets from these public structures and dynamics to our private phenomenal experience (and how there can be an equality between these opposite properties). This is known as the ‘structure and dynamics argument’ (Alter, 2016; Chalmers, 2003; Mindt, 2017), stating that structure and dynamics alone are not enough to account for consciousness. This raises a concern about whether any physicalist theory can solve the hard problem of consciousness. In the next sections, however, we will show that physicalism is broader than describing only structures and dynamics, and that we can use this fact in order to solve the hard problem.

Views about the relation between phenomenal and functional consciousness exist across a spectrum. On one end, illusionists seek to erase the hard problem by referring only to functional consciousness, taking phenomenal properties to be cognitive illusions. On the other end, naturalistic dualists or panpsychists seek to promote phenomenal consciousness as a fundamental, non-material, and undecomposable constituent of nature (Chalmers, 2017). The controversy over phenomenal consciousness can be traced to one central problem: naturalization. The project of naturalization involves taking folk psychological concepts and subjecting them to physical laws and empirical scrutiny (Hutto, 2007). Illusionists take the current scientific approach to consciousness and argue that this eliminates the messy problem with supposedly private, immaterial qualia. According to them, functional consciousness generates an illusion of special phenomenal properties, which create the persistent “user illusion” (Dennett, 1991) of a first-person perspective. Dualists start from the same problem of naturalization, but take it that phenomenal consciousness is simply not amenable to third-person scientific inquiry due to its sui generis properties. What is needed, according to the naturalistic dualist, is an expanded understanding of what counts as “natural.” “Given that reductive explanation fails, *nonreductive* explanation is the natural choice” (Chalmers, 2017, p. 359). Chalmers proposes that consciousness is a fundamental property, something like the strong nuclear force that is irreducible to other forces. A complete ontology of the natural world simply must include phenomenal consciousness as a basic, undecomposable constituent.

### The Zombie Argument and the Paradox of Phenomenal Judgment

Chalmers (1996) discusses the logical possibility of a *zombie*, a being physically, cognitively, and behaviorally identical to a human, but lacking phenomenal consciousness altogether. One would think that such a creature would be dull, like a robot with basic automated responses, but this is not the case. Because a zombie is physically identical to a human, it means that it has the same cognitive system as us: a system that gets inputs from the environment, processes them, and creates behavior responses. In fact, there are no differences between the human and the zombie’s cognitive dynamics, representations, and responses. But, for the zombie, there is nothing that is like to do all these processes. According to Chalmers (1996), the zombie has phenomenal judgments. This concept is very important for our argument, so let’s examine it a bit. Phenomenal judgements are higher-order cognitive functions that humans and zombies have in common. Humans are aware of their experience and its contents and they can form judgments about it (e.g., when we think ‘There is something red’), then, usually, they are led to make claims about it. These various judgments in the vicinity of consciousness are phenomenal judgments. They are not phenomenal states themselves, but they are about phenomenology. Phenomenal judgments are often reflected in claims and reports about consciousness, but they start as a mental process. Phenomenal judgments are themselves cognitive acts that can be explained by functional aspects like the manipulation of mental representations. That’s why zombies also have phenomenal judgements. We can think of a judgment as what is left of a belief after any associated phenomenal property is subtracted. As a result, phenomenal judgements are part of the functional aspect of consciousness. As Chalmers puts it (1996, p.174):

“Judgments can be understood as what I and my zombie twin have in common. A zombie does not have any conscious experience, but he claims that he does. My zombie twin judges that he has conscious experience, and his judgments in this vicinity correspond one-to-one to mine. He will have the same form, and he will function in the same way in directing behavior as mine… Alongside every conscious experience there is a content-bearing cognitive state. It is roughly information that is accessible to the cognitive system, available for verbal report, and so on.”

In other words, phenomenal judgments can be described as the representations of a cognitive system that bear content about phenomenology (which are not necessarily linguistic representations. E.g., such representation can be a representation of the color of the apple). In this paper, we will identify functional consciousness with the creation of phenomenal judgments.

As a result of the zombie’s capacity to create phenomenal judgements, we reach a peculiar situation: The zombie has functional consciousness, i.e., all the physical and functional conscious processes studied by scientists, such as global informational access. But there would be nothing it is like to have that global informational access and to be that zombie. All that the zombie cognitive system requires is the capacity to produce phenomenal judgments that it can later report. For example, if you asked it if it sees a red rose in front of it, using information processing, it might respond, “Yes, I’m definitely conscious of seeing a red rose,” although it is ultimately mistaken and there is truly nothing that is like for the zombie to see that rose. In order to produce this phenomenal judgment, despite having no phenomenal consciousness, the zombie cognitive system needs representations and a central system with direct access to important information enabling it to generate behavioral responses. It needs direct access to perceptual information, a concept of self to differentiate itself from the world, an ability to access its own cognitive contents, and the capacity to reflect. Such a cognitive system could presumably reason about its own perceptions. It would report that it sees the red rose, and that it has some property over and above its structural and functional properties—phenomenal consciousness. Of course, this report would be mistaken. It is a paradoxical situation in which functional consciousness creates phenomenal judgments without the intervention of phenomenal consciousness—yet phenomenal judgments are purportedly about phenomenal consciousness. This paradox of phenomenal judgment (Chalmers, 1996) arises because of the independence of phenomenal consciousness from physical processes. The hidden assumption here is that consciousness is private. Consequently, it is not possible to measure it. It seems that one aspect of consciousness (physical, functional consciousness) can come without the other (phenomenal consciousness). For example, in IIT there is a variant of the phenomenal judgement paradox. According to IIT, there can be a cognitive system that will manipulate information and infer that it has phenomenal experience, that there is something that is like to be that cognitive system, yet it doesn’t have any consciousness because its neural network creates all these judgements in a feed-forward way, meaning that 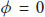 in the system (Doerig et al., 2019). Again, we see that even in IIT the functional part of consciousness (phenomenal judgements) can come without the phenomenal aspect of consciousness. This paradox arises because in IIT consciousness and cognitive content are not conditioned to correspond to each other. As a result, although the cognitive system has phenomenal judgements about phenomenal consciousness, still, it is just a zombie with no phenomenal consciousness because 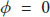 in the system (this kind of a zombie is termed functional zombie, see Oizumi et al., 2014; Tononi et al., 2016).

In order to solve this paradox, we need to explain two aspects of consciousness: How there could be natural phenomena that are private and thus independent of physical processes (or how come they *seem* private), and what the exact relationship between cognitive content and phenomenal consciousness is.

The illusionist position is that phenomenal properties are cognitive illusions generated by the brain. If a zombie with developed cognitive abilities can mistakenlythink it has phenomenal consciousness, how do we know that this is not the case with ourselves, as well? For the illusionist, this is exactly the predicament we are in, albeit we are zombies with a rich inner life (Frankish, 2017)—whatever that means. The illusionist takes the purported scientific intractability of phenomenal consciousness to be evidence against phenomenal consciousness. “Illusionists deny that experiences have phenomenal properties and focus on explaining why they seem to have them” (Frankish, 2017, p. 18). While this position might seem to be counterintuitive, it saves a conservative understanding of physics and obviates any call for exotic properties of the universe, as Chalmers (2017) argues for. As Graziano et al. (2020) state:

“‘I know I have an experience because, Dude, I’m experiencing it right now.’ Every argument in favour of the literal reality of subjective experience … boils down sooner or later to that logic. But the logic is circular. It is literally, ‘X is true because X is true.’ If that is not a machine stuck in a logic loop, we don’t know what is” (Graziano et al., 2020, p. 8).

The problem, however, is that it’s not clear what the claim that phenomenal consciousness is an illusion means. What exactly does “illusion” mean in this context? To be clear, the illusionist is not denying that you see, e.g., a red rose in front of you. You do “see” it, but it only *seems* like it has phenomenal properties. You have non-phenomenal access to the perceptual representation, sufficient to enable a phenomenal judgment about that representation (e.g., “I see a red rose”). This contention runs in the face of all our intuitions, but the illusionist claims those intuitions are illusory. Frankish (2017) states that these *seeming*-properties are quasi-phenomenal properties, which are physical properties that give the illusion of phenomenal properties. But are quasi-phenomenal properties any less mysterious than phenomenal properties? How is it that seeming to be phenomenally conscious is not just being phenomenally conscious?

One way to address these problems and understand the illusionist position is to understand them as agreeing with Chalmers about phenomenal consciousness’s privacy, and that zombies are logically possible. Then, the paradox of phenomenal judgment already notes that just such a functionally conscious cognitive system could produce phenomenal judgments without having phenomenal consciousness. However, illusionists take this to be evidence against phenomenal consciousness. A purely physical system creates phenomenal judgments, therefore there are nothing but purely physical processes involved (and hence, no qualia). However, this position is problematic. Only if phenomenal properties are private could there be such a paradoxical situation of having phenomenal judgments about phenomenal consciousness, yet without any phenomenal consciousness. If we can argue *against* the privacy of phenomenal properties, then we can escape the trap into which both the dualist and illusionist fall.

We interpret the dualist and illusionist extremes as unfortunate consequences of a mistaken view of naturalism. The illusionist’s commitment to naturalism leads them to exclude supposedly non-natural properties like qualia. The scientific dualist is also committed to naturalism, but takes it that the current inventory of nature is simply incomplete and that there must be a new, exotic fundamental property in the universe, that of phenomenality.

### The Relativistic Approach

A common thread connecting both extremes of dualism and illusionism is that both assume that phenomenal consciousness is an absolute phenomenon, wherein an object *O* evinces either property *P* or *^¬^P*. We will show that we need to abandon this assumption. The relativistic principle in modern physics posits a universe in which for many properties an object *O* evinces either property *P* or *^¬^P with respect to some observer X*. In such a situation, there is no one answer to the question of whether object *O* has property *P* or not. We propose a novel relativistic theory of consciousness in which consciousness is not an absolute property but a relative one. This approach eschews both extremes of illusionism and dualism. The relativistic theory of consciousness will show that phenomenal consciousness is neither an illusion created by a “machine stuck in a logic loop” nor a unique fundamental property of the universe. It will give a coherent answer to the question of the (supposed) privacy of phenomenal consciousness, will bridge the explanatory gap, and will provide a solution to the hard problem based in relativistic physics. General notions of this approach can be found in some dual aspect monisms such as Max Velmans’ reflexive monism. According to Velmans (2009, p. 298), “[i]ndividual conscious representations are perspectival.” Here, however, we develop a physical theory of consciousness as a relativistic phenomenon and formalize the perspectival relations in light of the relativistic principle. To do that, in the following section, we will develop more formally the *relativistic principle* and introduce the *equivalence principle of consciousness*.

In physics, relativity means that different observers from different frames of reference will nevertheless measure the same laws of nature. If, for example, one observer is in a closed room in a building and the other observer is in a closed room in a ship (one moving smoothly enough on calm water), then the observer in the ship would not be able to tell whether the ship is moving or stationary. Each will obtain the same results for any experiment that tries to determine whether they are moving or not. For both of them, the laws of nature will be the same, and each will conclude that they are stationary. For example, if they throw a ball toward the room’s ceiling, each will determine that the ball will return directly into their hands (because the ship moves with constant velocity and because of Newton’s First Law, the ball will preserve the velocity of the ship while in the air, and will propagate forward with the same pace as the ship. As a result, it will fall directly into the observer’s hands). There will be no difference in the results of each observer’s measurements, trying to establish whether they are stationary or not. They will conclude that they have the same laws of nature currently in force, causing the same results. These results will be the results of a stationary observer and thus both of them will conclude that they are at rest. Because each of them will conclude that they are the stationary one, they will not agree about one another’s status. Each of them will conclude that the other is the one that moves (common sense will tell the observer in the ship that they are the one moving, but imagine an observer locked on the ship in a room with no windows. Such an observer cannot observe the outside world. This kind of observers will conclude that they are stationary because velocity is relativistic).

To state that consciousness is a relativistic phenomenon is to state that there are observers in different *cognitive frames of reference*, yet they will nevertheless measure the same laws of nature currently in force and the same phenomenon of consciousness in their different frames. We will start with an equivalence principle between a conscious agent, like a human being, and a zombie agent, like an advanced artificial cognitive system. As a result of this equivalence, we will show that if the relativistic principle is true, then zombies are not possible. Instead, every purported zombie will actually have phenomenal consciousness and any system with adequate functional consciousness will exhibit phenomenal consciousness from the first-person cognitive frame of reference. Others have similarly claimed that zombies are physically impossible (Brown, 2010; Dennett, 1995; Frankish, 2007; Nagel, 2012), but our aim here is to show why that is according to the relativistic principle. As a result of this equivalence, observations of consciousness fundamentally depend on the observer’s cognitive frame of reference. The first-person cognitive frame of reference is the perspective of the cognitive system itself (Solms, 2021). The third-person cognitive frame of reference is the perspective of any external observer of that cognitive system. Phenomenal consciousness is only seemingly private because in order to measure it one needs to be in the appropriate cognitive frame of reference. It is not a simple transformation to change from a third-person cognitive frame of reference to the first-person frame, but in principle it can be done, and hence phenomenal consciousness isn’t private anymore. We avoid the term “first-person perspective” because of its occasional association with immaterial views of consciousness; cognitive frames of reference refer to physical systems capable of representing and manipulating inputs. These systems have physical positions in space and time and instantiate distinct dynamics.

In section “The Equivalence Principle of Consciousness: Mathematical Description,” we will show that from its own first-person cognitive frame of reference, the observer will observe phenomenal consciousness, but any other observer in a third-person cognitive frame of reference will observe only the physical substrates that underlie qualia and eidetic structures. The illusionist mistake is to argue that the third-person cognitive frame of reference is *the* proper perspective. To be clear, the first-person cognitive frame of reference is still a physical location in space and time (it is not immaterial); it is just the position and the dynamics of the cognitive system itself. As we will see, this is the position from which phenomenal consciousness can be observed. The principle of relativity tells us that there is no privileged perspective in the universe. Rather, we will get different measurements depending on the observer’s position. Since consciousness is relativistic, we get different measurements depending on whether the observer occupies or is external to the cognitive system in question. Both the first-person and third-person cognitive frames of reference describe the same reality from two different points of view, and we cannot prefer one point of view upon the other (Solms, 2021). The dualist mistake is to argue that phenomenal consciousness is private. For any relativistic phenomenon there is a formal transformation between the observers of different frames of reference, meaning that both frames can be accessible to every observer with the right transformations. Consciousness as a relativistic phenomenon also has such transformation rules. We will describe the transformations between first-person (i.e., phenomenological) and third-person (i.e., laboratory point of view) cognitive frames of reference. There are several consequences of these transformations. First, qualia and eidetic structures are not private. Rather, they only *appear* private, because in order to measure them one needs to be in the appropriate cognitive frame of reference, i.e., within the perspective of the cognitive system in question. We can use these transformations to answer questions like, “What is it like to be someone else?” Because of the transformations, results that we obtain from third-person methodology should be isomorphic to first-person structures. Isomorphism between two elements means that they have the same mathematical form and there is a transformation between them that preserves this form. Equality is when two objects are exactly the same, and everything that is true about one object is also true about the other. However, an isomorphism implies that everything that is true about *some* properties of one object’s structure is also true about the other. In section “The Equivalence Principle of Consciousness: Mathematical Description,” we will show that this is the case with measurements obtained from first-person and third-person frames of reference. We will show that this isomorphism is a direct result of the relativistic principle and the notion that phenomenal judgements and phenomenal structures are two sides of the same underlying reality. All that separates them are different kinds of measurements (causing different kinds of properties). An unintuitive consequence of the relativistic theory is that the opposite is also true, and first-person structures also bear formal equivalence to third-person structures. We advocate for interdisciplinary work between philosophers and cognitive neuroscientists in exploring this consequence.

## The Equivalence Principle of Consciousness

### The Principle of Relativity and the Equivalence Principle

Our task is to establish the equivalence principle of consciousness, namely, that qualitative and quantitative aspects of consciousness are formally equivalent. We start by establishing the equivalence between conscious humans and zombies, and then we expand that equivalence to all structures of functional consciousness. We begin with a philosophical defense of the equivalence principle and then develop a mathematical formalization. We must first present the principle of relativity and the equivalence principle in physics. Later, we will use these examples to develop a new equivalence principle and a new transformation for consciousness. To be more formal than earlier, the principle of relativity is the requirement that the equations describing the laws of physics have the same form in all admissible frames of reference (Møller, 1952). In physics, several relativistic phenomena are well-known, such as velocity and time, and the equivalence principle between uniformly accelerated system and a system under a uniform gravitational field. Let’s examine two examples with the help of two observers, Alice and Bob.

In the first example of a simple Galilean transformation, Alice is standing on a train platform and measures the velocity of Bob, who is standing inside a moving train. Meanwhile, Bob simultaneously measures his own velocity. As the train moves with constant velocity, we know that the laws of nature are the same for both Alice’s and Bob’s frames of reference (and thus that the equations describing the laws of physics have the same form in both frames of reference; Einstein et al., 1923/1952, p. 111). According to his measurements, Bob will conclude that he’sstationary, and that Alice and her platform are moving. However, Alice will respond that Bob is mistaken, and that *she* is stationary while Bob and the train are moving. At some point, Alice might say to Bob that he has an illusion that he is stationary and that she is moving. After all, it is not commonsensical that she, along with the platform and the whole world, are moving. Still, although it doesn’t seem to make common sense, in terms of physics all of Bob’s measurements will be consistent with him being stationary and Alice being the one who moves. In a relativistic universe, we cannot determine who is moving and who is stationary because all experiment results are the same whether the system is moving with constant velocity or at rest. Befuddled, Bob might create an elaborate argument to the effect that his measurement of being stationary is a private measurement that Alice just can’t observe. But, of course, both of their measurements are correct. The answer depends on the frame of reference of the observer. Yet, both of them draw mistaken conclusions from their correct measurements. Bob has no illusions, and his measurements are not private. Velocity is simply a relativistic phenomenon and there is no answer to the question of what the velocity of any given body is *without reference to some observer*. Their mistakes are derived from the incorrect assumption that velocity is an absolute phenomenon. As counterintuitive as it may seem, the relativity principle tells us that Bob is not the one who is “really” moving. Alice’s perspective is not some absolute, “correct” perspective that sets the standard for measurement, although we may think that way in our commonsense folk physics (Forshaw and Smith, 2014). Alice is moving relative to Bob’s perspective, and Bob is moving relative to Alice’s perspective. Furthermore, the fact that Alice and Bob agree about all the results from their own measurements means that these two frames of reference are physically equivalent (i.e., they have the same laws of nature in force and cannot be distinguished by any experiment). Because of this physical equivalence, they will not agree about who is at rest and who is moving.

Later, Einstein extended the relativity principle by creating special relativity theory. The Galilean transformation showed that there is a transformation between all frames of reference that have constant velocity relative to each other (inertial frames). Einstein extended this transformation and created the Lorentz transformation, which describes more accurately the relativistic principle. enabling us to move from measurements in one inertial frame of reference to measurements in another inertial frame of reference, even if their velocity is near the speed of light (Einstein, 1905; Forshaw and Smith, 2014). According to the transformation, each observer can change frames of reference to any other inertial frame by changing the velocity of the system. The transformation equation ensures that in each frame we’ll get the correct values that the system will measure. For example, the observer will always measure that its own system is at rest and that it is the origin of the axes. Indeed, this is the outcome of the transformation equation for the observer’s own system. Mathematically, a frame of reference consists of an abstract coordinate system, and (in tensor formulation) we denote each frame of reference by a different Greek letter (and by adding a prime symbol), usually by *μ* and *ν*, which are indexes for the elements of the vectors in each coordinate system. The Lorentz transformation is denoted by 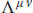. It is a matrix that gets elements of a vector in one frame of reference (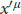) and gives back the elements of a vector in another frame of reference (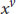):


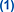



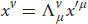


This equation coheres with the relativistic principle. That is, it describes the laws of physics with the same form in all admissible frames of reference. This form will stay the same regardless of which frame we choose. It allows us to switch frames of reference and get the measured result in one frame, depending only on the other frame and the relative velocity between the two.

The second example comes from Albert Einstein’s (1907) observation of the equivalence principle between a uniformly accelerated system (like a rocket) and a system under a uniform gravitational field (like the Earth). Here, Einstein extended the relativity principle even more, not only to constant velocity but also to acceleration and gravity (for local measurements that measure the laws of nature near the observer). He started with two different frames of reference that have the exact same results from all measurements made in their frames of reference. Then he used the relativity principle, concluding that because they cannot be distinguished by any local experiment they are equivalent and have the same laws of nature. Lastly, he concluded that because of the equivalence, we can infer that phenomena happening in one frame of reference will also happen in the other. Now, assume that Alice is skydiving and is in freefall in the Earth’s atmosphere, while Bob is floating in outer space inside his spaceship. Although Alice is falling and Bob is stationary in space, both will still obtain the same results of every local experiment they might do. For example, and if they were to release a ball from their hand, both of them will measure the ball floating beside of them (in freefall, all bodies fall with the same acceleration and appear to be stationary relative to one another—this is why movies sometimes use airplanes in freefall to simulate outer space). Although they are in different physical scenarios, both will infer that they are floating at rest. Einstein concluded that because they would measure the same results, there is an equivalence between the two systems. In an equivalence state, we cannot distinguish between the systems by any measurement, and thus a system under either gravity or acceleration will have the same laws of nature in force, described by the same equations regardless of which frame it is.

Because of this equivalence, we can infer physical laws from one system to the other. For example, from knowing about the redshift effect of light in accelerated systems with no gravitational force, Einstein predicted that there should be also a redshift effect of light in the presence of a gravitational field as if it were an accelerated system. This phenomenon was later confirmed (Pound and Rebka, 1960). According to Einstein (1911),

“By assuming this [the equivalence principle] to be so, we arrive at a principle which…has great heuristic importance. For by theoretical consideration of processes which take place relative to a system of reference with uniform acceleration, we obtain information as to the behavior of processes in a homogeneous gravitational field” (p. 899).

Next, we argue that phenomenal consciousness is a relativistic physical phenomenon just like velocity. This allows us to dissolve the hard problem by letting consciousness be relativistic instead of absolute. Moreover, we develop a similar transformation for phenomenal properties between first-person and third-person perspectives. The transformation describes phenomenal consciousness with the same form in all admissible cognitive frames of reference and thus satisfying the relativistic principle.

### The Equivalence Principle of Consciousness: Conceptual Argument

Before we begin the argument, it is essential to elaborate on what ‘observer’ and ‘measurement’ mean when applying relativistic physics to cognitive science. In relativity theory, an observer is a frame of reference from which a set of physical objects or events are being measured locally. In our case, let’s define a ‘*cognitive frame of reference*’. This is the perspective of a specific cognitive system from which a set of physical objects and events are being measured. Cognitive frame of reference is being determined by the dynamics of the cognitive system (for more details, see section “The Equivalence Principle of Consciousness: Mathematical Description”).

We use the term ‘measurement’ in as general a way as possible, from a physical point of view, such that a measurement can occur between two particles like an electron and a proton. Measurement is an interaction that causes a result in the world. The result is the measured property, and this measured property is new information in the system. For example, when a cognitive system measures an apple, it means that there is a physical interaction between the cognitive system and the apple. As a result, the cognitive system will recognize that this is an apple (e.g., the interaction may be via light and the result of it will be activation of retinal cells which eventually, after sufficient interactions, will lead to the recognition of the apple). In the case of cognitive systems, there are measurements of mental states. This kind of measurement means that the cognitive system interacts with a content-bearing cognitive state, like a representation, using interactions between different parts of the system. It is a strictly physical and public process (accessible for everybody with the right tools). As a result of this definition, the starting point of the argument is with measurements that are non-controversial, i.e., measurements that are public. These measurements include two types, measurements of behavioral reports and measurements of neural representations (like the phenomenal judgement-representations). For example, physical interaction of light between an apple and a cognitive system causes, in the end of a long process of interactions, the activation of phenomenal judgement-representation of an apple and possibly even a behavioral report (“I see an apple”).

Let us start from the naturalistic assumption that phenomenal consciousness should have some kind of physical explanation. The physicalism we assume includes matter, energy, forces, fields, space, time, and so forth, and might include new elements still undiscovered by physics. Panpsychism, naturalistic dualism, and illusionism all fall under such a broad physicalism. (It might be, for example, that physics hasn’t discovered yet that there is a basic private phenomenal element in addition to the observed known elements. If this element exists, it needs to be part of the broad physicalism.) In addition, let’s assume that the principle of relativity holds, i.e., all physical laws in force should be the same in different frames of reference, provided these frames of references agree about all the results of their measurements. Since we have accepted that consciousness is physical (in the broad sense), we can obtain a new equivalence principle for consciousness. Let’s assume two agents—Alice, a conscious human being—and a zombie in the form of a complex, artificial cognitive system that delusionally claims to have phenomenal consciousness. Let’s call this artificially intelligent zombie “Artificial Learning Intelligent Conscious Entity,” or ALICE. ALICE is a very sophisticated AI. It has the capacity to receive inputs from the environment, learn, represent, store and retrieve representations, focus on relevant information, and integrate information in such a manner that it can use representations to achieve human-like cognitive capabilities.

ALICE has direct access to perceptual information and to some of its own cognitive contents, a developed concept of self, the capacity to reflect by creating representations of its internal processes and higher-order representations, and can create outputs and behavioral responses (it has a language system and the ability to communicate). In fact, it was created to emulate Alice. It has the same representations, memories, and dynamics as Alice’s cognitive system and as a result, it has the same behavioral responses as Alice. But ALICE is a zombie (we assume) and doesn’t have phenomenal consciousness. On the other hand, conscious Alice will agree with all of zombie ALICE’s phenomenal judgments. After sufficient time to practice, ALICE will be able to produce phenomenal judgments and reports nearly identical to those of Alice. After enough representational manipulation, ALICE can say, e.g., “I see a fresh little madeleine, it looks good and now I want to eat it because it makes me happy.” It can also reflect about the experience it just had and might say something like, “I just had a tasty madeleine cake. It reminded me of my childhood, like Proust. There’s nothing more I can add to describe the taste, it’s ineffable.”

Alice and ALICE will agree about all measurements and observations they can perform, whether it’s a measurement of their behavior and verbalizations, or even an “inner” observation about their own judgments of their experience, thoughts and feelings. They will not find any measurement that differs between them, although Alice is conscious, and ALICE is not. For example, they can use a Boolean operation (with yes/no output) to compare their phenomenal judgments (e.g., do both agree that they see a madeleine and that it’s tasty?). Now, let’s follow in Einstein’s footsteps concerning the equivalence principle between a uniformly accelerated system and a system under a uniform gravitational field. Alice and ALICE are two different observers, and the fact that they obtain the same measurements agrees with the conditions of the relativity principle. Accordingly, because both of them completely agree upon all measurements, they are governed by the same physical laws. More precisely, these two observers are cognitive systems, each with its own cognitive frame of reference. For the same input, both frames of reference agree about the outcome of all their measurements, and thus both currently have the same physical laws in force. As a result, these two systems are equivalent to each other in all physical aspects and we can infer physical laws from one system to the other. According to the naturalistic assumption, phenomenal consciousness is part of physics, so the equivalence between the systems applies also to phenomenal structures. Consequently, we can infer that if Alice has phenomenal properties, then ALICE also must have them. Both Alice and ALICE must have phenomenal consciousness! ALICE cannot be a zombie, like we initially assumed, because their systems are physically equivalent. This equivalence makes it impossible for us to speak of the existence of absolute phenomenal properties in the human frame of reference, just as the theory of relativity forbids us to talk of the absolute velocity in a system. For, by knowing that there is phenomenal consciousness in the human frame of reference and by using the broad physicalist premise, we conclude that there are physical laws that enable phenomenal consciousness in the human frame of reference. Because of the equivalence principle, we can infer that the same physical laws will be present also in the supposed “zombie” cognitive system’s frame of reference. The conclusion is that if there is phenomenal consciousness in the human frame of reference, then the “zombie” cognitive system’s frame of reference must also harbor phenomenal consciousness.

We started from the premise that ALICE is a zombie and concluded that it must have phenomenal consciousness. One of our premises is wrong: either the broad physicalism, the relativistic principle, or the existence of zombies. Most likely the latter is the odd man out, because we can explain these supposed “zombies” using a relativistic, physicalist framework. As a result, although we started from a very broad notion of physicalism and an assumption that the human has phenomenal consciousness and the “zombie” cognitive system does not, the relativity principle forces us to treat phenomenal structures as relativistic. According to the relativity principle, there is no absolute frame of reference; there are only different observers that obtain different measurements. If the observers obtain the same measurements, there cannot be anything else that influences them. There is nothing over and above the observers (no God’s-eye-view), and if they observe that they have phenomenal properties, then they have phenomenal consciousness. Because there is nothing over and above the observer, we can generalize this result even further for every cognitive system that has phenomenal judgments: Any two arbitrary cognitive observers that create phenomenal judgment-representations also have phenomenal consciousness. Zombies cannot exist (assuming their cognitive systems create phenomenal judgments). In other words, we obtain an equivalence between functional consciousness (which creates phenomenal judgments) and phenomenal consciousness. Notice that even if we start from the naturalistic dualism of Chalmers and assume a broader physics including phenomenal elements alongside other aspects in the universe, the relativistic principle still forces this kind of broad physics to have the same consequences, viz., that zombies cannot exist and that there is an equivalence between phenomenal judgement-representations and phenomenal properties. (Formally, phenomenal judgement-representations and phenomenal properties are isomorphic. They have the same mathematical form and there is a transformation between them that preserves this form. See section “The Equivalence Principle of Consciousness: Mathematical Description”). As a result, the relativistic principle undermines the dualist approach altogether.

In the next section, we develop the mathematical proof of the argument. The mathematical description elaborates the fine details of the theory and reveals new insights. Notice that a mathematical background is not necessary to understand this section, as every step includes a comprehensive explanation.

### The Equivalence Principle of Consciousness: Mathematical Description

In this section we develop a mathematical description of consciousness as a relativistic phenomenon. In the beginning of the argument, we apply the relativistic principle only to publicly-observable measurements. Then, we show that we can also apply the relativistic principle to phenomenal properties and structures. To that end, we use two different arguments. Then, using the relativistic principle, we prove the equivalence principle between a conscious human agent and a purported “zombie.” Then, we expand that equivalence to all cognitive frames of reference having functional consciousness (i.e., the cause of phenomenal judgments). As a result, we prove that phenomenal judgements are equal to phenomenal properties in the cognitive frame of reference that generates them. Then, we describe the difference between the first-person and third-person perspectives and develop a transformation between them and between measurements of any cognitive frames of reference that have phenomenal consciousness. We show that this transformation preserves the form of the equation regardless of which frame we choose, and thus satisfies the relativity principle. That is, it describes the laws of physics with the same form in all admissible frames of reference.

#### The Three-Tier Information Processing Model for Cognitive Systems

Let’s once more assume two agents, Alice, a conscious human agent, and ALICE, a “zombie” artificial cognitive system (at least, supposedly) with phenomenal judgments. These systems parallel Chalmers’ thought experiment: ALICE is the cognitive duplicate of Alice, but lacks (we suppose) phenomenal consciousness. In order to develop a fully mathematical description, we need to know the exact equations of the cognitive systems in question. Unfortunately, to date there is no complete mathematical description of a human cognitive system, let alone an artificial one that can mimic human cognition. Instead, we use a general, simplified version, a mathematical toy model, to describe a cognitive system that creates phenomenal judgments. We use a three-tier information processing model that divides a cognitive system (*S*) into three parts: sensation (*T*), perception (*P*), and cognition (Dretske, 1978, 2003; Pageler, 2011). Sensation picks up information from the world and transforms it, *via* transduction, into neural signals for the brain. Later, perceptual states are constructed *via* coding and representations until a percept is created. The point at which cognitive processing is thought to occur is when a percept is made available to certain operations, such as recognition, recall, learning, or rational inference (Kanizsa, 1985; Pylyshyn, 2003). Cognition has many different operations, but our main interest is the module that specializes in functional consciousness (and that creates phenomenal judgments), which we label *C*. After recognition, this functional consciousness module needs to integrate the input and emotional information, the state of the system, the reaction of the system to the input, and self-related information in order to create representations of phenomenal judgments. We can summarize this model with the following equation:


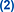



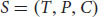


where *T* is the sensation subsystem, *P* is the perception subsystem, *C* is the functional consciousness subsystem of cognition, which is ultimately responsible for phenomenal judgments (we’re not considering other cognitive subsystems here). Finally, *S* is the entire cognitive system as a whole. These subsystems are a 3-tuple that form the cognitive system in a sequential manner. From *T* to *P* and to *C*. The three-tier model yields:


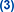



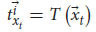



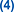



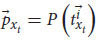



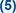



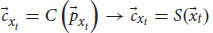


where 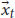 is an input from the physical space outside the cognitive system that interacts with the cognitive system at time *t*. The input has physical properties, *y_j_* (E.g., temperature, position, velocity, etc. *j* = 1,2,3… is an index of the properties). These physical properties can be detected by the sensors of the sensation subsystem (e.g., the input can be an apple and the cognitive system interacts with it by a beam of light that reached its retina at time *t*. Using photoreceptors in the retina, the sensation subsystem can detect physical properties of the apple like its color.) *T* is the sensation subsystem, and 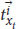 are the outputs of the subsystem, *i* = 1,2,3…n is an index for each sensory module (i.e., 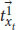 is the output vector of the sensation subsystem for visual information, 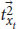 for auditory information, 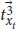 for interoception information etc.). Using the sensation subsystem, the cognitive system picks up information from the world and transforms it into neural patterns. Each pattern is a different possible state in the state space of the cognitive system. In this state space every degree of freedom (variable) of the dynamic of the system is represented as an axis of a multidimensional space and each point is a different state (e.g., the axes can represent firing rate, membrane potential, position, etc.). After a learning period a subset of these states is formed and being used by the sensation subsystem to encode inputs. This is the state space of the sensation subsystem, and it is part of the state space of the whole cognitive system. 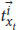 are vectors that point to such states in the state space of the sensation subsystem. There is a correspondence between the states 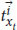 and the captured physical properties *y_j_* (e.g., correspondence between the color of an apple and the neural pattern that it causes in the system. light from a red apple activates photoreceptors which create a neural pattern. In this pattern, cones sensitive to red frequencies will fire more.) *P* is the perception subsystem. It gets 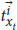 as inputs from the sensation subsystem and returns output 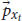, the percept of the input 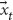. The perception subsystem creates representations. These are gists of important information about the input 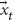 that the cognitive system can use even when the input is absent. the percept 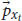 is a representation unifying information about the current input from all sensory modules. To that end, the perception subsystem using yet another subset of states from the state space. This is the state space of the perception subsystem, and it is part of the state space of the whole cognitive system. 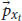 is a vector that points to such a state in the state space of the perception subsystem. There is a correspondence between the states and the operations that can act on these states and between the captured physical properties *y*_*j*_ (e.g., one operation on states can be addition of two states to get a third state in the state space of the perception subsystem. In our toy model, this operation can correspond to integration of different physical properties. A red round shaped object, for example, can be a state that is a summation of the red and round states in the state space of the perception subsystem.) *C* is the functional consciousness subsystem that creates phenomenal judgments (representations that are not phenomenal states themselves, but they are about phenomenology. see introduction for details). It gets percepts as inputs from the perception subsystem and returns phenomenal judgment representation, 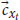 as output. As before, the functional consciousness subsystem using yet another subset of states from the state space. 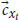 is a vector that points to such a state in the state space of the functional consciousness subsystem. There is a correspondence between the operations that can act on these states in the functional consciousness subsystem state space and between the captured physical properties *y_j_*. Combining these equations with eq. 2, the conclusion is that the cognitive system *S*, which uses the sensation, perception and functional consciousness subsystem sequentially, has a state space that is a combination of the three subsystems state spaces. It gets as input, 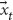 from the physical space, and returns as output, 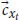, the phenomenal judgment representation, from the state space of the cognitive system. To sum, when Alice sees a red apple, for example, her perception subsystem creates a representation of sensory information, a unification of representations for “red,” “round,” and possibly a smell and texture. This integrated representation is her current percept. Later, the functional consciousness subsystem will recognize the object as an “apple” and will create an integrated representation of all relevant information about this red apple. This phenomenal judgment can be, for example, “good-looking, red, and roundish apple,” which can later be reported. Such a cognitive system *S* needs to integrate information from previous layers and from different modules. It needs an attention module (*W*) that can change the weights of representations and filter information to focus on relevant information. It needs a long-term memory module (*M*) to store and retrieve representations. It also needs an emotion evaluation module (*E*), which assesses information and creates preferences, rewards, and avoidances (e.g., does the input have a positive or negative affective valence?). Other modules could include an affordance module (*A*) that detects possibilities for action in the environment according to the input, and a self-module (*I*) that collects self-related information to create a unified representation of the self. Lastly, the cognitive system needs modules to create outputs and behavioral responses. In particular, it has a language module (*L*) that creates lexical, syntactic, and semantic representations and responses that the functional consciousness subsystem (*C*) can use. Adding them to Equations 4, 5, we obtain:


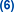



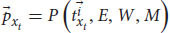


the perception subsystem P receives as variables the outputs of the sensation subsystem 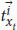 and uses the long-term memory module *M*, the attention module *W*, and the evaluation module *E* to create a percept 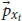 of the input 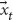, a unified representation of perception from all sensory modules. This yields:


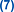



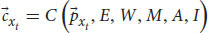


the functional consciousness subsystem *C* receives as a variable the output of the perception subsystem 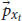 and uses the long-term memory module *M*, the attention module *W*, the evaluation module *E*, the affordance module *A* and the self-module *I* to create a phenomenal judgment 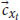 (a complex representation that carries content about phenomenology) concerning the input 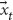. We can also add the language module for the cognitive system, so that *S* can understand and answer questions:


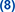



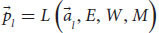


where *L* is the language module, 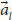 is the input of the language module, a low-level processed representation from the perception subsystem (without loss of generality, let us assume it to be auditory information. It can also be a low-level representation of other sensory modules). The language module L gets a low-level representation from the perception subsystem 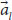 and uses the long-term memory module *M*, the attention module *W*, and the evaluation module *E* to create several representations. Semantic representations for words, syntactic representations, and eventually sentence comprehension representation, 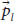. As before, the language module using yet another subset of states from the state space. 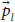 is a vector that points to such a state in the state space of the language module. The functional consciousness subsystem *C* uses the output of the language module to perform its tasks and create phenomenal judgments (answering a question about a red apple, for example):


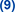



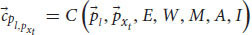


Now we add 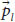 to Equation 7, which is the sentence comprehension representation (a question, for example) as another variable of *C*. The answer, 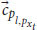, is sent to the language module, this time creating a linguistic response:


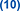



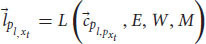


where *L* is the language module and 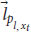 is a vector of the linguistic response. As before, the language module using yet another subset of states from the state space. 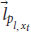 is a vector that points to such a state in the state space of the language module. This state is a complex neural pattern caused by motor neurons that innervate muscle fibers. The pattern causes contraction patterns in the muscles to create a linguistic response. The response is according to 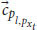, the phenomenal judgement representation that captures both the question and the answer of *C*. For example, suppose that Alice asks ALICE what she sees, and ALICE sees a red apple. The visual information will be transduced and processed in the perception subsystem until a percept is created (equation 6). The language system creates a representation of the question, 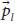 = “what do you see?” (eq. 8) and the functional consciousness subsystem uses the representation of the question, 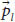 and the percept of the apple, 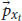, as variables to build a phenomenal judgement to answer the question, 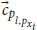 (eq. 9). Finally, the language module uses this representation to produce a proper linguistic response, 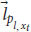 = “I see an apple” (eq. 10).

#### The Equivalence Principle of Consciousness

In parallel with Chalmers’ (1996) assumptions about zombies, Alice and ALICE have equivalent cognitive systems. But Alice, being human, also has phenomenal consciousness (without loss of generality, let’s assume Alice has a quale 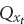 about input 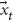. We will not assume any inner structure for the quale). After we establish the equations for the cognitive system, we can use them to prove the equivalence principle for consciousness. Let’s start with general considerations about cognitive frames of reference. A cognitive frame of reference is determined by the dynamics of a cognitive system (represented by Equations 1–10). Because the equations create and use representations, the representations are an inseparable part of the dynamics of a cognitive system. If two cognitive systems have different dynamics and representations, they are in different cognitive frames of reference. But if they have the same dynamics and representations, then they are in the same cognitive frame of reference. We will denote cognitive frames of reference by *μ*, and *ν*.

We started with the assumption that ALICE is a zombie. Hence, ALICE will obtain the same behavioral reports (i.e., 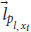) and the same phenomenal judgments (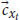) as Alice, who also has phenomenal consciousness (note that for this claim to be true, we idealize the situation as if Alice and ALICE were standing in the same spatial position. Hence, they will obtain the same phenomenal judgments. We can neglect minor differences arising from their different spatial positions, as long as they are standing near each other). In fact, Alice and ALICE have the same exact cognitive structure and dynamics (i.e., the same subsystems, modules, dynamics, and representations). As a result, they are in the same cognitive frame of reference and for each question they will have the same responses and the same phenomenal judgments. We can formalize it as such:


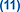



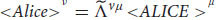


where *ν* is Alice’s cognitive frame of reference, *μ* is ALICE’s cognitive frame of reference, and < > is a symbol for the set of all results of measurements of a system (i.e., < Alice > means the set of results of measurements conducted by Alice). 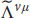 is a general transformation from the set of results of measurements of frame *μ* to the corresponding set of results of measurements of frame *ν*. Alice and ALICE can measure or observe both their behavioral reports and their phenomenal judgments, and then compare the results. They will discover that their results are the same. For the same input, they will not find any measurement about themselves to distinguish between them (again, the fact that they stand in slightly different spatial positions can be neglected because any reasonable cognitive system takes different positions into account in order to build percepts correctly, regardless of the system’s position. For example, the cognitive system needs to recognize an apple regardless of the angle between the apple and the system. Objects should be invariant structures across changing conditions.) According to the relativistic principle, because they obtain the same results about themselves, they have the same physical laws currently in force in their respective frames of reference. There is an equivalence between the two systems, and we can infer physical laws and phenomena from one system to the other. For example, we know that


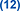



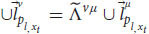


There is a transformation from the unification of all linguistic responses of Alice, 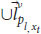, to the unification of all linguistic responses of ALICE, 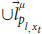 (provided that both were asked the same questions, 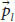). Let’s consider a specific linguistic response. From Equation 11 we obtain:


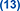



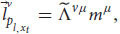


where *m^μ^* is a specific measurement result of ALICE, *m^μ^* ∈ < *ALICE* >. 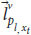 is a specific linguistic response of Alice. There is a transformation from the linguistic response of Alice that she will measure (i.e., that she will observe about herself) in her cognitive frame of reference to a specific measurement that ALICE will obtain about herself in her frame of reference. Now we can ask, what is the specific measurement in ALICE’s frame of reference? Because of the relativistic principle we know that the frames of reference of Alice and ALICE are equivalent, and hence we just need to replace *ν* with *μ* :


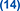



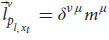



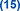



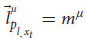


We use a special function known as the delta function, 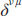, to transform from frame *ν* to frame *μ*. This function equals 0 if *v* ≠ *μ*, and equals 1 if *v* = *μ*. In other words, the only solution that is different from 0 for Equation 14 is when *v* = *μ*. Now we can plug in 1 instead of 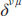 and substitute *μ* for *ν* on the left-hand side of Equation 15, with the result that ALICE will measure 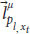. Now we can plug the result of Equation 15 back in Equation 13, which gives us a similar equation to Equation 12 for a specific linguistic response. If we repeat this process for every linguistic response of the two systems, we obtain Equation 12 (for details, see Supplementary Note 1). Proceeding in this manner allows us to infer physical laws and new phenomena from an observer in one frame of reference to the observer in the other frame. Next, we will use this process to infer the existence of qualia in ALICE’s frame of reference (similar to what Einstein did with the equivalence principle between uniform acceleration and uniform gravitational field). The transformation term 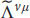, is equal to the delta function because of the relativistic principle. Because Alice and ALICE obtain the same measurement results, they are in the same cognitive frame of reference.

So far, we have applied the relativistic principle only to public measurements like reports and phenomenal judgements. But we know that Alice also observes quale 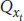 in her frame of reference. According to broad physicalism, phenomenal consciousness has some kind of physical explanation (physical laws that govern phenomenal consciousness) and as such it is part of physics and part of the physical measurements that Alice can conduct in her first-person frame of reference, *ν* (even if it’s a unique, private measurement). But for the relativistic principle to hold true, all physical laws currently in force in both respective frames of reference should be the same (including the physical laws that enable qualia). Thus, because of our broad physicalist assumption, we can obtain from Equation 11:


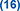



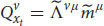


where 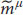 is yet another specific measurement result of ALICE and 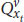 is Alice’s quale about input *x*_*t*_. Just as before, there is a general transformation between the quale that Alice measures in her frame of reference to a specific measurement that ALICE observes about herself in her own frame of reference. Because of the relativistic principle and the equivalence between the two frames of reference, all we need to do is replace *ν* with *μ*:


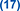



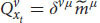



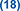



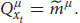


We see that the relativistic principle yields that ALICE will also measure a quale in her frame of reference (and thus zombies cannot exist). We can plug Equation 18 back into Equation 16 and repeat this process for all Alice’s qualia. As a result, Alice and ALICE will measure the same qualia about themselves (just like they obtain the same behavioral response and the same phenomenal judgments, assuming we can neglect the small differences due to their differing spatial positions).

This proof leaves the possibility that even though zombies cannot exist because of the relativistic principle, phenomenal consciousness can still be private. To accommodate that, we need to develop yet another mathematical argument, one that is more detailed (eq. 19-35). In addition, the second argument doesn’t use the assumption of broad physicalism (at least not explicitly). Notice that until now we haven’t used the equations of the cognitive systems. Let’s go back to Equation (13), for the linguistic reports:


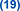



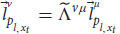


From equation (10) about the linguistic module, we obtain:


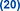



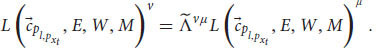


We can obtain similar equations for the functional consciousness sub-system (from Equation (9)):


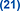



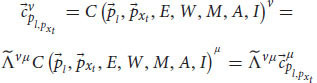


Now, consider a special case where Bob asks ALICE and Alice a question about the experience they just had (for example, 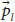 = “What are you experiencing right now?”). In this case, the cognitive system needs to check the phenomenal judgment representation itself. As a result, the functional consciousness subsystem will have the last phenomenal judgments as an input, 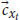. Plugging it into Equation 9 gives:


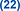



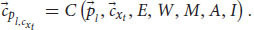


The answer, 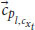, is yet another phenomenal judgment representation concerning the previous phenomenal judgment and the question. The linguistic module will use this representation to create a linguistic response according to Equation 10:


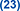



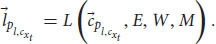


The linguistic response can be, e.g., 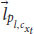 = “I’m experiencing happiness right now.” In this scenario we can write the same transformation between ALICE and Alice for the functional consciousness subsystem and the linguistic module as before:


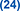



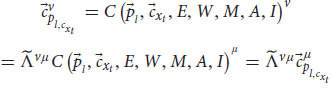



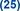



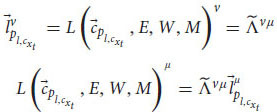


For all these transformations in Equations 19–21, 24–25, we can substitute the general transformation term, 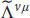, with a delta function, because of the equivalence between Alice’s and ALICE’s frames of reference (both have the same cognitive system and measurements):


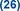



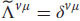


For example, Equations 24–25 will get the form:


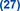



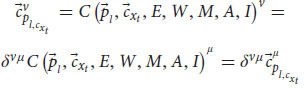



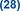



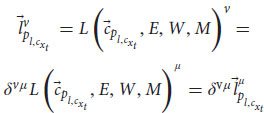


Now, let’s switch to Alice’s frame of reference. From Alice’s point of view, she has phenomenal consciousness. This perspective is also a physical frame of reference. From her first-person perspective, she experiences happiness (quale 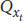) and a question, *Q*__*l*__ = “What are you experiencing right now?” After the question, she will have an experience of an answer to the question, 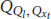, and accordingly she will respond with 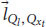 = “I’m experiencing happiness right now.” Notice that from her first-person frame of reference, she directly experiences her qualia and uses the quale (e.g., of happiness) to answer the question (in her head) and to articulate the answer as a linguistic report. We can formulate an equation according to her first-person frame of reference:


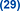



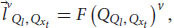


where 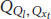 is the quale of the answer, 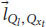 is the linguistic response, and *F* is a general function mapping a quale to a vector of the linguistic response. From Alice’s frame of reference, she experiences herself trying to answer the question in her head 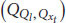 and then responds accordingly 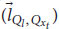. Her direct experience is that her linguistic response, 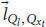 is a result of the quale of her answer, 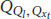.

We can now make use of the equivalence between Alice and ALICE. First, notice that we can describe Alice’s linguistic response according to her first-person perspective (Equation 29) and according to a third-person perspective (Equation 23). Both descriptions refer to the same linguistic response, and thus 
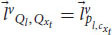
. Plugging this result into Equation 29 gives us:


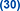



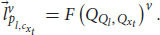


According to Equation 28, because of the equivalence between Alice’s and ALICE’s frames of reference for linguistic responses, we can replace the left-hand side of Equation 30 with:


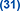



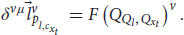


Because of the delta function, we know that *ν* = μ, and we can substitute *μ* with *ν* on the right-hand side of Equation 31:


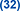



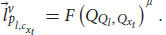


We have thus completed the transformation to ALICE’s frame of reference. ALICE, like Alice, must have qualia from her first-person perspective, which she uses to create a linguistic response. As a result of Equation 32, we notice again that the relativistic principle yields that ALICE, as cognitive frame of reference *μ*, will also observe a quale in her frame of reference. This invalidates Chalmers’ (1996) assumption that a zombie can be physically and computationally isomorphic to a phenomenally conscious person (which we assumed in the beginning by stating that ALICE was a zombie). Notice that now we can use Equation 23 and substitute 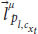 in Equation 32 with:


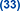



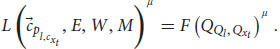


There is an equivalence between the form of the right-hand side of the equation and the form of the left-hand side of the equation (in both sides there is a function that gets input). We can identify the function *F* as the function of the linguistic module, *F = L*, and the input of function *F* as the input of function *L* (notice that the modules *E,W, M* are not the inputs of *L*, they are just modules that the dynamics of the language module uses to create the linguistic response according to the input 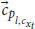):


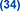



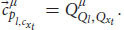


We here get an interesting result in ALICE’s frame of reference. Not only will ALICE observe a quale just like Alice, but this quale is exactly her complex integrated representation that bears content about phenomenology (‘phenomenal judgement’). We can identify between the inputs 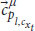 and 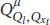 because in conscious agents like humans there is a close relation between the two. In fact, there is an isomorphism between them, and we can do a one-to-one correspondence mapping between them. As we saw, every quale will always be accompanied with a corresponding phenomenal judgment and every phenomenal judgment will always be accompanied with a corresponding quale. This pair of quale and phenomenal judgment is unique for each input 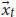 and every time 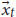 is present for the conscious cognitive system, if one part of the pair is present then the other part will be present as well. We saw that zombies cannot exist because they must have qualia according to the relativistic principle (eq. 32). We also saw that in the cognitive frame of reference that uses phenomenal judgments there is an equality between the functions that use qualia and phenomenal judgments (eq. 33). The identity between the functions and the isomorphism between qualia and phenomenal judgments are enough to show that in the cognitive frame of reference that uses phenomenal judgments there is an equality between qualia and phenomenal judgments (eq. 34). We reach an equivalence between phenomenal consciousness and phenomenal judgments that were created by cognitive systems, and hence obtain an equivalence between functional consciousness and phenomenal consciousness. It is easy to show that if we use the equivalence principle once again to move back to Alice’s frame of reference, we will obtain the same result for her, as well. We developed this equation from a special case of a quale about a quale (the experience of an experience, for example, 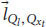 = “I’m experiencing happiness right now.”), but we can repeat the same process for different cases of qualia, such as a quale of an arbitrary input (seeing an apple for example), 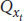. Generally, from the first-person perspective, a quale causes something to happen, whether it will cause a linguistic response (like in Equation 29) or the next associative thought. Because there is always an equivalent phenomenal judgment representation that will do the same thing in the cognitive system, we can always repeat a similar process like we did here (Equations 29–34) with an adequate equation similar to Equation 29 for the specific case. Furthermore, we can use the identity in Equation 34 in these similar equations to prove similar identities for different kinds of qualia (see Supplementary Note 2 for details). The relativistic principle ensures that because we cannot distinguish between the quale and the phenomenal judgment representation that accompanies it, they are the same thing. Consequently, we can generalize Equation 34:


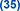



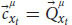


In the first-person frame of reference, there is an equality between the quale and the phenomenal judgment-representation, and hence the quale is a point in an appropriate state space and can be represented as a vector. The first-person frame of reference is the frame of the cognitive system measuring itself. In other words, in its dynamics, the cognitive system uses its own representations as inputs and outputs and they interact with each other (like in our cognitive system model, Equations 1–10).

#### Conditions for Third- and First-Person Frames of Reference

What happens when we move to different cognitive frames of reference? Why can’t they directly measure the quale of the other frame of reference? Why is there a difference between first-person and third-person perspectives, if it’s not because of some phenomenal property of privacy? To answer these questions, let’s expand the equations beyond the simple case of the equivalence between a conscious human and a copy of the human’s cognitive system (a zombie-like system). We showed that in the simple case of Alice and ALICE, not only are both cognitive systems equivalent, but they are also in the same cognitive frame of reference, *v* = *μ* (that’s why we could use the delta function).

Now, let’s examine two cognitive frames of reference that are not a copy of each other: Alice and Bob. These two cognitive systems developed separately and there is no reason to assume that they learned to associate inputs with the same neural patterns. Each associated different states as representations from the state space according to its own personal developmental history. As a result, Alice and Bob have no states in common and the set of stats that Alice’s cognitive system uses is disjoint with the set of states that Bob’s cognitive system uses. As before, from her first-person frame of reference, Alice has an experience of an apple (quale 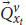). What will happen if Bob tries to directly observe the quale of Alice in his cognitive frame of reference? We can describe this process from his cognitive frame of reference, *μ*. His perception, functional consciousness, and linguistic subsystems (Equations 6–8) directly obtain Alice’s quale:


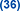



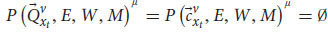



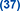



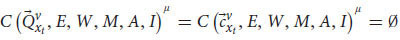



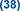



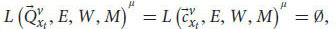


where 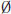 is the empty set. To measure Alice’s quale directly means to use it directly in Bob’s cognitive frame of reference. Because her quale is equal to a phenomenal judgment representation (Equation 35), we substitute 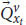 with 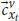. But because Bob and Alice are two different cognitive frames of reference, Bob’s cognitive system doesn’t recognize Alice’s representation, and we get an empty set. As a result, Bob cannot directly measure Alice’s quale. The only solution for Bob is to indirectly measure it using his sensation subsystem (Equation 3).

What will Bob measure while using his sensation subsystem to measure Alice’s quale? Recall that the sensation subsystem measures physical properties, *y*_*j*_ from outside of the cognitive system, that can be detected by its sensors. According to eq. 35, qualia, like phenomenal judgments, are neural patterns or states of the state space of Alice’s cognitive system (that are being measured directly in Alice’s cognitive frame of reference). every degree of freedom (variable) of the dynamics of the system is an axis of this space (e.g., the axes can be firing rate, membrane potential, position of firing neurons, etc.). The axes form the basis vectors of the state space, 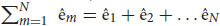 where 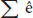 means a summation over the basis vectors and m = 1,2,3…N is an index that runs from 1 to N, according to the N-dimensions of the state space. Each state can be written, in a unique way, as 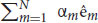, where α_1_…α_*N*_ are coefficients. These Numbers are the coordinates of the axes (e.g., suppose that for the neural representation of an apple there is a specific firing rate. This is the coordinate α_*1*_ of the first axis ê_1_, the axis of firing rates and we will denote it by, α_1_ê_1_.) These variables and coefficients of the dynamics of Alice’s cognitive system are the physical properties *y_j_* that Bob’s sensation subsystem will measure in the case of Alice’s quale/representation, 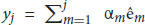 (e.g., without losing generality, let’s assume that Alice’s quale/phenomenal judgement while seeing an apple will have a specific pattern of 3 variable: firing rates, membrane potentials and position of firing neurons. Each of them with a specific coefficient. Bob’s sensation subsystem will measure these 3 properties with their specific coefficients.):


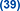



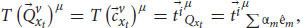


where 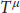 is Bob’s sensation subsystem, 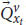 is Alice’s quale, 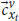 is Alice’s phenomenal judgment representation, 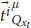 are the outputs of the sensation subsystem of Bob about Alice’s quale. 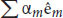 are the variables and coefficients of Alice’s quale/representation, and 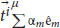 are the outputs of the sensation subsystem of Bob about the physical properties of Alice’s quale. Bob’s sensation subsystem is the only part of the cognitive system that can measure Alice’s quale, though indirectly. As before, we substitute her quale with the corresponding representation. The sensation subsystem can measure the physical properties of the representation and create an output that Bob’s cognitive frame of reference can utilize (e.g., a visual sensation of a brain pattern). Only then will Bob’s cognitive frame create its own percept, and eventually a quale of this sensation. Notice that this quale is about the indirect measurement of Alice’s quale, a brain pattern that Bob measured from Alice’s brain:


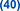



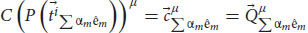


Notice the difference between Alice’s initial quale, 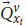, and Bob’s final quale about her brain pattern, 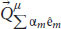. Interestingly, even in the same cognitive frame of reference (of Alice, for example), if we introduce to Alice’s sensation subsystem one of Alice’s qualia, the sensation subsystem will still not recognize it directly as a quale. Instead, the subsystem will measure the physical properties corresponding to that quale, 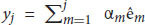. For example, we can show Alice an activation pattern going on in her brain as she reports seeing an apple. Alice will not recognize this pattern as her representation of seeing an apple and will instead see a visual image of the brain pattern. This is because the sensation subsystem still doesn’t use representations, let alone the representations of higher cognitive subsystems like perception and cognition. It just measures physical properties from outside of the cognitive system:


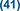



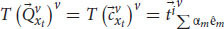


Only the parts of the cognitive system that use the phenomenal judgment representation, 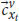, will measure it directly (such as the functional consciousness subsystem and the language module). The relativistic principle ensures us that this direct measurement of a phenomenal judgment representation is a quale (Equation 35). Any other cognitive frames of reference will just measure the physical substance of the representation *via* the sensation subsystem. It seems that the reason we have such a distinct difference between first-person and third-person perspectives is due to the direct measurement of the phenomenal judgment representation in the functional consciousness subsystem, and the function of the sensation subsystem that specializes in measuring bare physical properties and not representations. In sum, the condition for the ability to measure a quale (viz., the first-person frame of reference) is to have a subsystem that uses the corresponding phenomenal judgment representation (i.e., to measure directly phenomenal judgment. Because then the functional consciousness subsystem uses the phenomenal judgment in its dynamics and according to eq. 35, we can substitute it with the appropriate quale):


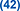



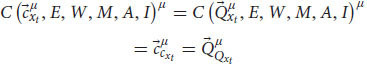


Here, the functional consciousness subsystem, 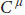, uses in its dynamics (directly measures) a phenomenal judgment representation, 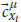, and hence measures it directly to create a new phenomenal judgment representation about it, 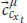. According to the relativistic principle, these phenomenal judgments are measured as qualia, 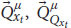. The condition to have a third-person frame of reference is to activate the sensation subsystem (39, 41):


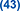



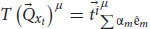


Notice that the cognitive frame of reference that measured the quale is irrelevant for the sensation subsystem. The output will always be the same: sensations of the physical properties of the quale/phenomenal judgement, 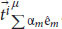.

#### Transformation Between Cognitive Frames of Reference and Between First- and Third-Person Frames of Reference

Finally, according to the principle of relativity, all admissible frames of reference measure the same laws of physics that are currently in force, and hence the equations describing the laws of physics have the same form in all admissible frames of reference (like Equation 1, describing the Lorentz transformation between all inertial frames of reference). As a relativistic phenomenon, consciousness should also have a similar transformation that preserves the same form of the transformation equation for all admissible cognitive frames of reference. Let’s develop this equation. The transformation should take us from the measurements of one frame, *μ* (Bob), to the measurements of another frame, *ν* (Alice). Regardless of the measurements, the equation should look the same (for example, if one of the measurements causes new terms to appear in the equation, the form of the equation is no longer the same, and thus the equation is not relativistic because we can distinguish between the measurements according of their different terms. As a result, the equivalence between the admissible cognitive frames of reference has broken. We need to ensure that this is not the case in our equation). Notice that, in contrast to the scenario of Alice and ALICE, now the transformation has no constraints in the form of a question that guides the observers. Instead, they measure the same input, and the transformation should give the answer for what the cognitive system in each frame of reference measures. When we move from measurements of a quale in one cognitive frame of reference, *μ*, to measurements of a quale in a second cognitive frame of reference, *ν*, there are three cases of what they can measure. The cognitive frames can measure a third input (e.g., an apple), they can measure frame *μ*, or they can measure frame *ν*. If they measure a third input, like an apple, each of the frames will have its own quale about the apple, 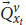, 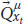. If they measure the quale of frame *ν*, then frame *ν* measures the quale 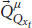 directly, while frame *ν* will measure the physical properties of the quale of frame *μ*, 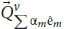, and vice versa if they measure the quale of frame *ν*.

We need to use another transformation term, 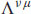, for this general scenario. It needs to give the correct measurements for each case, while preserving the form of the transformation equation. Because we lack the accurate equations of the cognitive system and use general forms in the equations, the transformation equation will also be in a general form:


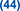



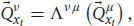


where 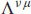 is the transformation function from cognitive frame μ to cognitive frame *ν*, and 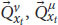 are the qualia of input 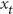 as measured in frames *ν*, μ respectively.

In order to build 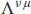, for the transformation to operate successfully, one key element is to check the physical properties, *y*_*j*_ of the input. To that end we introduce the function *m_C_* that checks if the physical properties *y*_*j*_ of the input 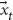 are the physical properties of the representations that the functional consciousness subsystem uses as outputs, 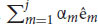. In other words, it checks whether the input is a state of the state space of the functional consciousness subsystem *C* (i.e., phenomenal judgments). If it does then *m_C_* returns the state 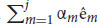 and if the input is not part of the state space of *C*, then *m_C_* returns the empty set Ø:


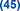



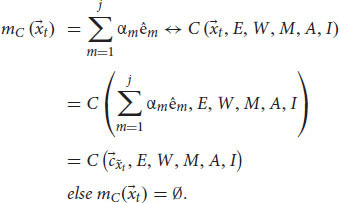


If the input 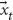 is a state of the state space of *C* then it’s a phenomenal judgment, 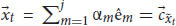 where 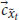 is a phenomenal judgment about some input 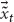. In that case, *m_C_* returns this state. Notice that because *C* can get phenomenal judgments as inputs (see eq. 22 and 42) 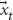 can be input of C and thus can be measured directly by the functional consciousness subsystem. If the input is not part of the state space of *C*, then *m_C_* returns 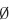. Now we can introduce the transformation function 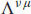:


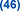



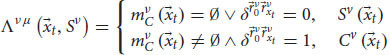


Where ∨ is a symbol of the logic operation ‘or’ and Λ is a symbol of the logic operation ‘and.’ In order to carry out the transformation from cognitive frame *μ* to cognitive frame *ν*, the transformation function 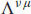 gets as inputs the input 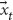 and cognitive system of frame *ν*, *S^ν^*. 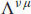 checks two conditions, whether 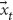 is a state of the state space of *C^ν^* (the functional consciousness subsystem of frame *ν*), and whether 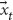 is in 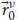, the spatial position of cognitive system *S^ν^* (as seen from its own cognitive frame of reference, *ν*). The spatial position of 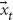 as seen from cognitive frame of reference *ν*, is 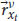. Spatial positions 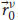, 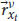 are part of the physical properties of the cognitive system of frame *ν* and the input (respectively). The transformation function checks the first condition by plugging 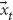 into 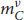 (that checks whether the input is a state of the state space of *C* in the cognitive frame of reference *ν*) and checks the second condition by using a delta function, 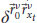, that equals 1 if 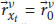 and 0 if 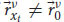. If 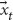 is not a state in the space state of *C^ν^*, then 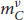 will return the empty set. This means that the input 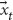 cannot be measured directly by the functional consciousness subsystem of frame *ν*. The same situation occurs if the delta function is equal to 0, it means that 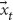 is not in the cognitive system’s spatial position and thus is not available for *C^ν^* to use it and measure it directly. As a result, 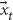 is sent as an input to cognitive system *S^ν^* for an indirect measure. There, the sensation subsystem of frame *ν* gets 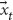 as input. From there, the process will continue along the hierarchy of the cognitive system to the perception subsystem and the functional consciousness subsystem of frame *ν* (see eq. 2, 5). If 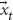 is a state in the state space of *C^ν^*, then 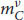 will not return the empty set. This means that the input can be measured directly by the functional consciousness subsystem of frame *ν*. In addition, the second condition is being checked and if the delta function is equal to 1, it means that the input is in the spatial position of the cognitive system and thus available for *C^ν^*. As a result, if the two conditions are fulfilled, 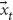 is sent as an input directly to *C^ν^*, the functional consciousness subsystem of cognitive system S of frame *ν*, to measure the input directly as a quale. In sum, cognitive system *S* of frame *ν* is in the third-person perspective if either 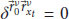 or 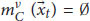 because it uses the sensation subsystem (condition for third-person frame of reference is fulfilled, eq. 43) and cognitive system S of frame *ν* is in the first-person perspective only if both 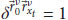 and 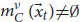, because then it uses *C^ν^* (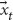) which measures directly the phenomenal judgment (i.e., a quale. Condition for first-person frame of reference is fulfilled, eq. 42).

Let’s check if Equation 46 gives the correct measurements for each case of the transformation from frame *μ* to *ν* while preserving the form of the transformation equation (Equation 44). We mentioned three cases: the cognitive frames can measure a third input, (e.g., apple), they can measure frame *μ*, or they can measure frame *ν*. In the first case, we start from a quale of frame *μ* about a third input like an apple, 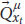. The transformation function checks if the input 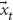 is a state from the state space of *C^ν^* or is it inside the cognitive system *S^ν^*. Because this is an input from outside of the functional consciousness subsystem and the cognitive system, 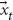 (the apple) is not a state of the subsystem and not in the spatial position of the cognitive system, and hence the transformation function sends 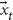 to *S^ν^*, the cognitive system in frame *ν*. As a result, *S^ν^* will use its sensation, perception, and functional consciousness subsystems to create its own quale of the apple, 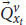. The transformation function succeeds in giving the correct output for the first case. In the second case, frame *μ* measures its own quale 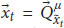 and thus in the transformation equation (eq. 44), 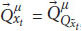. The transformation function checks if the input 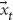 is a state from the state space of *C^ν^* and if it is part of the cognitive system *S^v^*. Because this is again an input from outside of the cognitive system and the functional consciousness subsystem of frame *ν* (it’s a quale from frame *μ*), the transformation function sends the input to *S^ν^*. As a result, frame *ν* will use its sensation, perception, and functional consciousness subsystems to create a quale of the physical properties of the quale from frame *μ*, and thus in the transformation equation (eq. 44) 
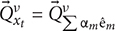
. The transformation function succeeds in giving the correct output for the second case as well. In the last case, frame *μ* measures a quale 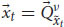 of frame *ν*. As a result, frame *μ* will measure the physical properties of the quale from frame *ν*, and thus in the transformation equation (eq. 44), 
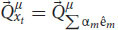
. The transformation function checks if the input 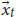 is a state from the state space of *C^ν^* and if it’s part of the cognitive system *S^ν^*. This time, the answer is positive for both conditions. Because this is an input from within the functional consciousness subsystem of frame *ν*, the matching function of the subsystem 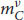 will return something other than the empty set and the delta function will be equal to 1. Consequently, the transformation function sends 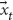 directly to the functional consciousness subsystem of frame *ν* [where, 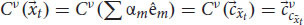, see Equation 45]. As a result, frame *ν* will measure its own quale, and thus in the transformation equation (eq. 44) 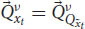. The transformation function also succeeds in giving the correct output for the last case. Notice that the transformation Equation 44 maintains the same form regardless of the specific measurement case or which cognitive frames of reference are involved. Hence, Equation 44 satisfies the relativity principle as desired.

All the preceding scenarios can give the impression that the two conditions are redundant. They always seem to agree with each other and so, one condition is enough to get the correct transformation. But now let’s choose a special case where *v* = *μ*. Hence, the transformation function will be 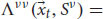 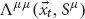. There are two scenarios for this case. One scenario is the identity transformation in which the transformation leaves us in the same cognitive system that we started from (and thus in the same cognitive frame of reference). According to Equation 46, 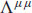 gives the correct answer in this scenario, because we stay in the same cognitive system all the time (same frame and same spatial location). In other words, the transformation function doesn’t change anything, 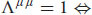

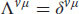
. We can call this the full symmetry scenario, because the two cognitive frames are completely symmetric and we cannot distinguish between them (in other words, we started from a cognitive system and come back after the transformation to the same system). The transformation Equation 44 will be in this scenario,


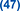



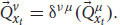


The second scenario, however, is more complicated. Here we have two cognitive systems but they are in the same cognitive frame of reference. They have the same dynamics and thus the same representations and the same qualia, but they remain two discrete systems with different spatial positions. As a result, for the same input both will have the same answer to every question they might be asked. This is the scenario that we started from when we developed the equivalence principle between ALICE and Alice. It’s very special scenario where we have two cognitive systems in different spatial positions but in the same cognitive frame. Notice, however, that even though they are in the same cognitive frame, because they have different spatial positions, they cannot measure directly the representations of the other system and thus they must use their sensation subsystem and measure indirectly the quale of the other system (similar to Equation 41 where the same cognitive system will still measure the physical properties of its own quale due to the sensation subsystem). In other words, although they are in the same cognitive frame of reference, Alice will measure ALICE from a third-person perspective and vice versa. Will Equation 46 give the correct transformation from frame *μ* (ALICE) to *ν* (Alice) also in this scenario?

For the first case, both Alice and ALICE measure a third input like an apple. In this case, when we transfer from ALICE to Alice, we will get an answer just like in the previous, full symmetric scenario. Because the physical properties of the apple is from the outside of the cognitive system *S^v^*, the transformation function will use the sensation, perception, and functional consciousness subsystems of the system in frame *ν* to create the appropriate quale, 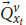. But because both systems are in the same cognitive frame, their qualia are the same and the quale of the input will remain the same. As a result, the transformation gives us the correct answer for this case,


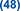



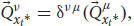


where 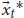 is input from outside of both cognitive frames. ALICE and Alice will measure the same quale about an input that is outside of both their cognitive frames.

If ALICE (*μ*) measures its own quale, 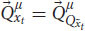, the transformation function checks if the input 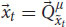 is a state from the state space of *C^ν^* and if it’s in the position of the cognitive system *S^ν^*. In this case, because 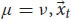 is a state of the functional consciousness subsystem of frame *ν* as well (the state 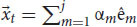 is the same state in both frames). But the other condition fails because the systems are not in the same spatial position, and 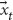 comes from the position of the other cognitive system, *S^μ^*. As a result, 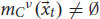, but 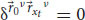. Because only one condition is fulfilled for the first-person perspective and not both, the transformation function will send 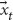 to *S^ν^* for an indirect measure. From the sensation subsystem in frame *ν* until eventually creating a quale of the physical properties of the quale from frame μ, 
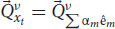
. The transformation function succeeds in giving the correct answer also for this case. If ALICE (*μ*) tries to measure the quale of Alice (*ν*) the result will be a quale of the physical properties of Alice’s quale, 
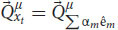
. The transformation function checks if the physical properties of the input 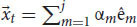 is a state from the state space of *C^ν^* and if it’s in the position of the cognitive system *S^ν^* of frame *ν* (Alice). This time, the answer is positive for both conditions. Because the input is also a state from the state space of the functional consciousness subsystem of frame *ν* and it is in the position of *S^ν^*, the matching function of the subsystem 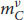 will return something other than the empty set and the delta function will be equal to 1. Consequently, the transformation function sends 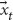 directly to the functional consciousness subsystem of the cognitive system in frame *ν*. As a result, frame *ν* will measure its own quale, 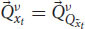. The transformation function also succeeds in giving the correct output for the last case. Now we see that the last two cases in the scenario of ALICE and Alice are different than the full symmetry scenario. The different spatial positions of the cognitive systems break the symmetry of the identity transformation. Although the systems are in the same cognitive frame, the different positions cause the transformation to be different than 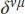. The different spatial positions break the symmetry because they cause the cognitive systems to use their sensation subsystem to measure the quale of the other system. We can call this scenario the broken symmetry scenario. As a result, we can write the transformation Equation 44 for this scenario as:


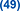



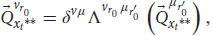


Which gives us -


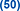



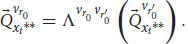


Here 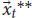 is an input of a state from the state space of *C* either from frame *ν* or μ in the broken symmetry case. The delta function is presented because both cognitive systems are in the same frame, but 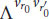 is still different than the identity transformation because the symmetry of the cognitive systems was broken by their different spatial positions, 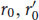 (for another example of the importance of the spatial positions condition, see Supplementary Note 3). Because of the broken symmetry scenario, we define the first-person frame of reference slightly differently than the definition of a cognitive frame of reference. The first-person frame of reference is defined not only by the dynamics of the cognitive system but also by its position. As a result, Both Alice and ALICE will have the same answers to the question of what is it like (because they are in the same cognitive frame of reference), but because they are not in the same spatial position, they are not in the same first-person frame of reference. When one is measuring the dynamics of the other, all they see is the physical patterns of the other cognitive system. In other words, when one cognitive frame is in another spatial position, it has to use its sensation subsystem and thus satisfy the condition for the third-person frame of reference (eq. 43). Only if the two identical cognitive systems can be in the same position can they share the same dynamics and use their functional consciousness subsystem directly without the need to use the sensation subsystem. Only then will they satisfy the condition of the first-person frame of reference (eq. 42).

Now we also see that the general transformation, 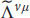, that we used for the equivalence principle in order to prove eq. 35 is a special case of the transformation function we developed here, 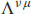. In this case, the two cognitive frames of reference are the same but have different spatial positions (e.g., Alice and ALICE), and their input is constrained to be a question (the same question for both cognitive frames of reference). Because both frames are the same and because their input is the same question, we always get a scenario like eq. 48 and thus, 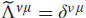.

The conclusion is that the transformation function 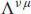 gives the correct answers to all cases and scenarios while preserving the form of the transformation equation (equation 44) as desired. Equation 46 for 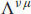 can be approximated by a simpler form:


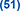



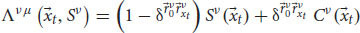


The first term is the condition to be in a third person perspective because of the use of the sensation subsystem. Only when 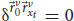 will this term be equal to 1. The second term is the condition of being in the first-person perspective (because of the use of the functional consciousness subsystem). Only when 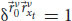 will this term be equal to 1. This approximation checks only if the spatial position of the input is the same as the spatial position of the cognitive system and it gives the correct answers to all scenarios. It transforms from the third-person frame of reference to the first-person frame of reference and vice versa. But it is only an approximation because it does not take into account the matching function *m*_*C*_(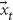) and so in the case of two cognitive systems from the same cognitive frames of reference (like Alice and ALICE) the approximation will neglect this information and the result will be two different cognitive frames ν, μ instead of just one.

In Supplementary Note 4, we show the inverse transformation function, from frame of reference *ν* to frame *μ* and that the transformation function and the inverse transformation function cancel each other out (giving the identity transformation), as expected.

## Discussion

### Consciousness as a Relativistic Phenomenon

As we saw, the assumption that the relativity principle also includes measurements of cognitive systems forces us to treat consciousness as a relativistic phenomenon. If the relativity principle is true, then zombies are not nomologically possible (see Equations 18, 32). In other words, they are not consistent with the extant laws of nature (note that this is a different claim from logical possibility). Recall that both naturalistic dualism and illusionism can be understood to be opposing responses to the same basic paradox of phenomenal judgment: phenomenal judgments seem to not require phenomenal consciousness. This opens the possibility for zombies. Chalmers (2017, 1996) takes the threat of zombies to force us to accept that phenomenal properties are an additional fundamental component of reality. Illusionists instead take *us* to be the zombies (Frankish, 2017; Graziano et al., 2020). But we have demonstrated that zombies are not nomologically possible in a relativistic universe. The illusionist can, of course, point out that we have assumed that Alice is phenomenally conscious, which they do not grant. While this is an assumption (and not a controversial one for any but the illusionist), what we point out is that the illusionist contention that there are zombies, and that *we* are the zombies, is premised on the paradox of phenomenal judgment. But we have eliminated that paradox by eliminating the nomological possibility of zombies. Therefore, we have eliminated the illusionist motivation to deny that Alice is phenomenally conscious. While we can’t prove that Alice is indeed phenomenally conscious, the illusionist no longer has a reason (viz., the paradox of phenomenal judgment) to doubt it.

As a result of the relativity principle, there is a formal equivalence between functional consciousness (creating phenomenal judgments) and phenomenal consciousness (qualia and eidetic structures; see Equation 35). Ultimately, we are returning to Nagel’s (1974) definition of consciousness: that the creature is conscious if there is something that is like to be this creature. If an observer has phenomenal judgments, then the observer measures that there is something that it is like to be that observer, and this observer is conscious. The essence of the relativistic principle is that there is nothing over and above the observer. Hence, in contrast to illusionism and naturalistic dualism, there cannot be any third-person or God’s-eye perspective telling us that some observer is delusional and doesn’t *really* have consciousness.

Moreover, phenomenal properties are no longer absolute determinations, but depend on the observer’s cognitive frame of reference, just as in the case of constant velocity and the question of who’s stationary and who’s moving. In a non-relativistic universe, an object *O* evinces either property *P* or *^¬^P*. Properties are absolute determinations. But in a relativistic universe, an object *O* evinces either property *P* or *^¬^P with respect to some observer X*. In the first-person cognitive frame of reference, quale *Q* (for example) is observable, while any third-person cognitive frame of reference observes *^¬^Q*, i.e., that there is no quale.

Cognitive frames of reference are defined by the dynamics of the cognitive systems involved (according to the three-tier information processing model, eqs. 2–10). The first-person frame of reference takes into account also the position of the dynamics (eqs. 49–50). The first-person frame of reference of Alice, for example, is the position from within Alice’s cognitive system, which is a physical position in space and time where the dynamics of the system take place. This is the only situation that satisfies the condition for the first-person frame of reference (eq. 42). For that reason, the transformation function 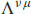 (that transforms between cognitive frames of reference and between first and third frames of reference) takes into account not only whether the input is recognizable by the cognitive system as one of its own representations, but also if the input is in the position of the cognitive system (eq. 46). The third person frames of reference are the positions of other cognitive systems, like Bob, that measure Alice’s cognitive system. While Alice may observe herself feeling happiness as she’s looking at a rose, Bob will only measure patterns of neural activity. Recall the case of constant velocity, wherein Alice claims to be at rest while Bob is moving, while Bob claims that Alice is the one moving and that he is stationary. From Alice’s perspective, she has qualia and Bob only has patterns of neural activation, while from Bob’s perspective Alice just has patterns of neural activation while he has qualia. In other words, just as in the case of constant velocity, Bob and Alice both measure that they are the stationary ones, and hence will not agree on who is stationary and who is moving, there is likewise a relativistic equivalence between all cognitive systems that have phenomenal consciousness or functional consciousness. Both Alice and Bob will measure that they are the ones who have phenomenal consciousness while the other has only neural patternsand hence will not agree on who has phenomenal consciousness.

Alice and Bob will continue to argue over who is right. As a result, Alice might be an illusionist and claim not only that Bob is delusional about having qualia, but moreover that qualia don’t exist at all. And, as a response, Bob might claim that qualia are uniquely private and non-physical phenomena. But, just as with the constant velocity case, their conclusions are wrong because they don’t grasp the relativistic principle. The illusionist mistake is to claim that the third-person perspective is the only legitimate perspective. When Alice observes Bob’s neural firing, he supposedly should infer that all his “qualia” really are just neural firing. Yet the first-person perspective is also a physical arrangement of a cognitive system. It is the cognitive system from its own observer perspective. Both frames of reference are equivalent, there is no observer position that is privileged, and it is impossible to privilege one observer cognitive position over the other. Alice’s perspective is not some absolute, “correct” perspective that sets the standard for measurement. Bob doesn’t have phenomenal consciousness relative to Alice’s perspective but *does* have phenomenal consciousness relative to Bob’s perspective. In other words, Bob evinces *^¬^Q* with respect to Alice, and Bob evinces *Q* with respect to Bob. This is not a logical contradiction, but a statement of the relativity of properties.

On the other hand, qualia and eidetic structures are not private and hence phenomenal consciousness is neither some mysterious force beyond the realm of science nor an irreducible element of reality. Rather, they *appear* to be private because in order to measure them, one needs to be in the appropriate frame of reference, viz., that of the cognitive system in question (see Equations 42, 45). It is ultimately a question of causal power. Only from this frame of reference is there causal power for the representations in the dynamics of the system. Only from the frame of reference of the cognitive system are these neural patterns recognized as representations (eq. 45). These representations are the input and output of the cognitive system (eq. 2-10); they are the ones that cause the dynamics in this cognitive frame of reference. From outside that observer reference frame, as in the position the neuroscientist takes as a third-person observer, the same exact phenomena appear as neural computations. This is because the third-person perspective is constitutively outside of the dynamics of representations of the cognitive system in question, and hence these representations do not have any causal power over the neuroscientist (Equations 36–38). According to the equivalence principle, when Alice observes herself to be happy, it is because her cognitive system can recognize and use the appropriate phenomenal judgments, and these judgments are equivalent to phenomenal consciousness (Equation 35), because there is always an equivalent phenomenal judgment representation for every phenomenal property, and we cannot distinguish between them. These representations and their relations cause the cognitive dynamical system to react with new representations and instantiate new relationships between them. As a result, we get a dynamical system that uses *very specific* representations as variables and as outputs. Any other cognitive system, like that of Bob, uses *different* representations and thus cannot use the representations of Alice directly in its own dynamics (Equations 36–38). The only possibility left for Bob is to process Alice’s representations through his sensory system and build his own representations. Consequently, we get a sharp difference between the self-measurements of the cognitive system (first-person perspective) and measurements from the outside the cognitive system (third-person perspective), which are mediated solely through the three-tier hierarchy—from the sensation subsystem to the perception subsystem and on to the functional consciousness subsystem (Equations 39, 40). Ultimately, the reason for the sharp difference between first-person and third-person frames of reference is because the sensation subsystem can observe only the physical properties (y_*j*_) of inputs from the outside of the cognitive system, while the functional consciousness subsystem can only observe representations from within the cognitive system (Equations 41–43). Even if the two cognitive systems at hand are in the same cognitive frame of reference (which mean that they have the same dynamics), because they have different spatial positions where their dynamics take place, they will not be in the same first-person frame of reference. They will still need to use their sensation subsystem to measure the other system (hence satisfying the condition of the third-person frame of reference, eq. 43).

Phenomenal properties are not truly private. They seem private because it is non-trivial to do the transformation to the appropriate cognitive frame of reference. Nevertheless, this kind of transformation is nomologically possible. We showed that, as a result of the relativity principle, there is a transformation between the qualia of all cognitive frames of reference (Equations 44, 46, 51). Using this transformation, we obtained an equation that agrees with the relativistic principle (Equation 44). It describes the laws of physics with the same form in all admissible cognitive frames of reference. In other words, this form will stay the same regardless of which cognitive frame we choose (it doesn’t matter for the equation what the specific *μ* and *ν* cognitive frames are). The equation enables us to move from the phenomenal consciousness of one cognitive frame of reference to the phenomenal consciousness of another frame and from first-person frame of reference to third person frame of reference (and vice versa). According to the transformation, each observer can change cognitive frames of reference to any other frame by changing the dynamics of its cognitive system. The transformation equation is built in such a way that ensures that after we applied the transformation and moved from one frame to the other, we will get the correct values of the measurements in the new cognitive frame of reference. For example, the equation ensures that the relations between first- and third-person frames of reference are always satisfied. The observer will always measure qualia and eidetic structures from within its own frame, and brain patterns (or any other physical patterns that govern the cognitive system) for all other frames. The only way to enter the frame of reference of the cognitive system is to have the dynamics of representations of that system, because they only have the right kind of causal power from within that cognitive system (they are the right “fuel” of the cognitive system – the inputs and outputs of the system). Hence, third-person studies will get different measurements than those taken from within the system itself, unless a proper transformation were to change the frame of reference of the third-person cognitive system to have the exact dynamics of the cognitive system in question (see Equations 36–38 for third-person frame, in contrast to Equations 42, 45 for the first-person frame). After such a transformation, there would be an equivalence between the two systems and they would observe the same conscious experience. Obviously, no such transformation is currently technologically feasible, but it is nomologically possible. Because of this transformation that preserves the form of the measurements, phenomenal structures (first-person perspective) and phenomenal judgement-representations (third-person perspective) are equivalent and isomorphic to each other. They are not equal, but they have the same preserved mathematical form such that we can map between them. All that separates them are different kinds of measurements (causing different kinds of properties).

### The Hard Problem Dissolves

The relativistic theory of consciousness dissolves the hard problem. If there is no irreducibly private property of phenomenal features, there is no need for adding new, exotic elements to reality, nor is there a need to explain phenomenal features away as illusory. There is also no explanatory gap, because there is no need to explain how physical patterns in the brain create private, irreducible properties. Because consciousness is a relativistic phenomenon, physical patterns (e.g., of neural representations) and phenomenal properties (e.g., qualia) are two sides of the same coin. Both are valid physical descriptions of the same phenomenon from different frames of reference. Although phenomenal properties supervene on physical patterns (the representations that we called ‘phenomenal judgements’), they are not created by the physical patterns. Instead, phenomenal properties are the result of a special measurement of these physical representations by the observer. For this observer, these representations are the ones that cause its own dynamics. Notice that according to the relativistic theory of consciousness, the opposite is also true and physical patterns (phenomenal judgements) supervene on phenomenal properties. They are not created by phenomenal properties; instead, physical representations are the result of a measurement of these phenomenal properties by a different observer. For this cognitive frame of reference, these phenomenal properties have no causal power over its dynamics. Phenomenal and physical aspects supervene on each other. There is a subtle identity between them (i.e., they are equivalent). They are just different perspectives of the same phenomenon from different cognitive frames of reference. As a result, the relativistic theory of consciousness is a physicalist theory, but not a reductive theory, because there is no reduction of phenomenal properties to brain patterns. For phenomenal properties we need a cognitive system with the right kind of representations and the right kind of measurement.

In the introduction, we raised a concern about whether any physicalist theory can solve the hard problem of consciousness. How can there be an identity between public properties like structure and dynamics and between private qualia? The answer is that we didn’t include relativity as a part of physicalism. The relativistic theory of consciousness shows that phenomenal states are not private and gives an explanation of why they are different from other physical states. Different kinds of measurements give rise to different properties. While our sensation subsystem can only directly measure a physical substrate via measurement devices like the retina, the perceptual and cognitive subsystems directly measure only the roles and relations of representations within their subsystems and cannot directly measure the physical substrate serving as the referent of their representations (that’s why in the equations that describe the dynamics of the perceptual and cognitive subsystems, the inputs are representations and not the physical substrate of the representations, while in the sensation system the inputs are the physical substrates themselves like light or sound waves). From these different kinds of measurements different kinds of properties arise.

Chalmers (1996, 2017) parses the zombie argument in terms of the logical possibility of zombies. He would not deny that zombies are inconsistent with the extant laws of nature, because he speaks only about logical possibility in general. However, the relativistic theory of consciousness carries ontological constraints and conditions about the existence of consciousness in every possible world. For example, the difference between first- and third-person frames of reference arise because of the difference between the kind of measurements that can be taken within each frame of reference. There could be a possible world, not governed by the known laws of nature, wherein the relativistic principle is false. But then, there would be no different frames of reference, and the different kinds of measurements would yield the same result. In other words, there would be no first-person and third-person frames of reference. In such a possible world, we would be left with either the illusionist view, with no consciousness at all (i.e., everybody is a zombie with just third-person perspective), or we would be left with a Berkeleian idealist world where there are only phenomenal properties (with shared first-person perspective for everything). Consequently, according to our relativistic theory of consciousness, there cannot be a world that has both phenomenal consciousness *and* zombies. This conclusion undermines the zombie argument. The main goal of the zombie argument was to establish that phenomenal properties cannot be explained reductively in terms of physics. But the relativistic theory does just that, demonstrating that phenomenal consciousness and physics can be reconciled. There might be a logical possibility of zombies, but in such possible worlds, the physical mechanism that allows the transformation to phenomenal properties (i.e., the relativistic principle) must be absent and hence consciousness must be absent all together as well (because all there is left in such a world is the physical substance that constitutes the world). In sum, in non-relativistic worlds without different frames of reference, we could have *either* illusionism (only physical properties) *or* Berkeleian idealism (only phenomenal properties). But in a relativistic world where different frames of reference measure different properties, *both* physical *and* phenomenal properties are possible for the same entity.

Phenomenal consciousness is not private—all we need to do to measure phenomenal properties is to change our perspective by moving to the appropriate frame of reference, i.e., the frame wherein the representations have causal power (i.e., the frame that measures the representations directly). As a result, this frame doesn’t measure them merely as physical patterns, but as what they represent and stand for. For example, the representation of an apple has some causal power in the system, according to what has learned about apples. The representation will trigger, for example, memories, emotions, and motor processes. For the cognitive system, this is what it means to be an apple. Thus, the physical properties of the representation are not being directly measured, but rather only its role and relations with other representations in the system. Consequently, this representation will be measured by the system as an apple and not as a representation of an apple. That is to say, because there is nothing above and beyond the observer, and because the cognitive system (the observer) measures this representation according to what it does (the representation describes all the functions, properties and relationships of an apple in the system), this representation will be measured by the system as an apple. (That’s why phenomenal consciousness is characterized by transparency, i.e., we don’t perceive representations but directly perceive things). This is a direct measure of the representation itself. In the case of phenomenal judgments representations, these new properties are being measured as phenomenal properties. Specifically, when there is a cognitive frame of reference that creates functional consciousness (phenomenal judgments), it is sensible to assume that such a system will have a special subsystem specializing in such complex representations (the functional consciousness subsystem, *C*). It uses the complex representations of functional consciousness for its dynamics (i.e., 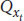 – phenomenal judgment representation, see eq. 5). As a result, these representations have causal power in this frame of reference. The relativity principle ensures us that because no measurement can distinguish between phenomenal properties and their corresponding phenomenal judgments, this cognitive frame will measure these phenomenal judgments as qualia and eidetic structures (Equation 35). In sum, according to the relativistic theory of consciousness, for phenomenal structures we need a cognitive system with the right kind of representations and the right kind of measurement. Phenomenal structures are the direct measurements of the complex representations that we called phenomenal judgments.

The relativistic theory of consciousness suggests a solution for the hard problem based in relativistic physics. There are still several open questions that need to be addressed in the future. For example, what are the necessary and sufficient conditions for a cognitive frame of reference to have phenomenal consciousness? And what empirical predictions does this theory yield? On the philosophical side, there are also questions about the affinities and differences between our view and dual aspect monisms such as reflexive monism (Velmans, 2009). These are issues that we will address looking forward.

### The Formal Equivalence of Phenomenal and Neurocomputational Structures

Since consciousness is relativistic, there is a formal equivalence between first-person phenomenological structures and third-person neurocomputational structures. Philosophers have asked and puzzled over the question, “What is it like to be a bat?” (Nagel, 1974). Nagel’s point is that physicalism is not sufficient for explaining consciousness. Even if we describe the neural processes involved in bat sonar, we will never get an intuitive or imagistic sense of what it is like from the first-person perspective. Yet years of philosophical arguments about the impossibility of a complete scientific understanding of consciousness has only stymied serious research, relegating consciousness to something that is supposedly not measurable and hence not amenable to scientific treatment (Holland, 2020).

By the relativistic theory of consciousness, the neurocomputational structures are equivalent to the phenomenal structures of what it is like to be a bat. This equivalence is not a statement of the reductive physicalism that Nagel attacks. We do not maintain that phenomenology can be reduced to neural computation. Rather, we maintain that phenomenology and neural computation are two different ways that the same phenomenon appears based on the cognitive perspective of the observer. That perspective is either from within the cognitive system (the first-person perspective) or outside of the cognitive system (the third-person perspective). This equivalence allows us to use neurocomputational structure to derive phenomenal structure. In other words, phenomenal consciousness can be investigated by studying neurocomputational dynamics. Certainly, the only way that the intuitive, imagistic aspects of “what it is like” could be perceived is by actually taking the cognitive frame of reference of the system in question. But qualia and eidetic structures can be measured indirectly through the neural phenomena they manifest from the third-person cognitive frame of reference. Phenomenal consciousness is not an immaterial and extra-scientific phenomenon, but one that’s amenable to the scientific method (for an example of a similar approach, see Petitot, 2018).

Not only can neurocomputational structure help us discover phenomenological structure. Furthermore, phenomenology can also help us to discover neurocomputational structures themselves. We are referring to neurophenomenology (Varela, 1999; Gallagher, 2003; Varela and Thompson, 2003; Petitmengin, 2006; Petitot, 2018; Gallagher and Zahavi, 2020). Rigorous first-person philosophical inquiry into phenomenal structure can help guide cognitive neuroscience research into consciousness by tracing the lineaments of what scientists ought to be looking for. While descriptions of phenomenal consciousness are often thought of as wispy and vague, there are precise ways that phenomenological philosophy (and so-called “cognitive phenomenology”) describes phenomenal consciousness that go beyond naïve, folk conceptions of experience.

For the illusionist, who understands phenomenal consciousness to be a cognitive illusion of phenomenal properties, “cognitive scientists should treat phenomenological reports as fictions” (Frankish, 2017, p. 27). In the relativistic perspective, however, phenomenological reports are not fictions. Of course, there are serious methodological issues that make data collection from the first-person perspective the purview of philosophy rather than science (Husserl, 1925/2012). But phenomenal reports are reports of what the system is like from within its own cognitive frame of reference. These reports can potentially help guide cognitive neuroscientific investigation. Since consciousness is relativistic, phenomenal structure is simply a different way of perceiving the same phenomenon that the cognitive neuroscientist is examining from a different observer perspective. Rather than denigrating the first-person perspective, we believe the relativistic framework invites us to take phenomenological philosophy seriously. We believe it poses potentially rich possibilities for interdisciplinary work on consciousness. No doubt, such interdisciplinary work would be fraught with difficulties, but the phenomenological philosopher looking at phenomenal consciousness and the cognitive neuroscientist looking at functional consciousness are both looking at the same thing from different observer positions.

Let’s get a clearer idea on what philosophers can contribute to the empirical study of consciousness. Typically, first-person experience is spoken of in terms of qualia, which often picks out the content of phenomenal consciousness: what it is like to see red (Jackson, 1982), or what it is like to be a bat and have sonar (Nagel, 1974). But the tradition of phenomenological philosophy, initiated by Edmund Husserl and continued by Martin Heidegger and Maurice Merleau-Ponty, has long studied first-person experience in terms of eidetic structures. An eidetic structure refers to the “form” or “essence” (*eidos*) of a given phenomenon in first-person experience, that is, its invariant structure across time. Phenomenologists are interested not in the qualia of greenness, but in the structural invariants that are expressed in all phenomenal consciousness, such as intentionality or the temporal structure of cognition and consciousness. We will close this section by describing the eidetic structure of what phenomenologists call time-consciousness (Husserl, 1917/1991; Neemeh and Gallagher, 2020). We will hypothesize a way that this eidetic structure may be realized as a neurocomputational structure. This should be understood as an invitation for the kind of interdisciplinary exploration between philosophers and cognitive neuroscientists that we have advocated for.

The subjective experience of time has a formal eidetic structure, what Husserl (1917/1991) calls time-consciousness. While we usually imagine time to be a linear arrow, phenomenologists contend that time-consciousness has a far more complicated, looping structure (Neemeh and Gallagher, 2020). Take the perception of a melody. How can you perceive a melody if, at any given point in time, you only hear a single note being played? Don’t you simply perceive individual notes? But a series of individual notes is not the same as hearing a melody. The eidetic structure of melody perception involves what phenomenologists call protention and retention. First of all, the immediate present is never a simple, non-extended slice of time. The present of experience is a “specious present” (James, 1890/1983) that is temporally extended. When one note is perceived in the immediate present, it recedes into the immediate past. Yet it is still retained, in an attenuated form, in consciousness (called “retention”). It is retained not as a sensuously fulfilled object, but as a trailing shadow (Husserl, 1917/1991). Only the immediate now is fully sensuous. But if past notes were not retained in this way in consciousness, we would not have a conscious perception of a melody. We would only hear individual notes. At the same time, melody perception involves an anticipation (or protention) of the imminent note. This is how we can perceive a wrong note: it doesn’t match up with our anticipation, based on the previously given aspects of the melody. The same principle holds when we hear a sentence.

In the relativistic view, first-person experience is no longer ineluctably private. It is simply one specific cognitive frame of reference, and when we switch perspectives, by the appropriate transformation (eq. 44, 46, 51), to that of cognitive neuroscience (third-person perspective), we ought to (ideally) detect isomorphic structures that are not qualitative but rather quantitative (assuming our phenomenological analysis is correct, of course).

Given this formal equivalence (Equation 44), there is a neurocomputational structure that is equivalent to the eidetic structure of time-consciousness, or perhaps there are multiple such neurocomputational structures for time-consciousness in different sensory modalities. Cognitive neuroscientific investigation can use this eidetic structure as a guide to inquiry. For example, Varela (1999) suggests an account based in phase locking, and Neemeh and Gallagher (2020) suggest a Bayesian approach.

Philosophers have long studied phenomenal consciousness and may have practiced insight into what structures to look out for in functional consciousness. This is not to say that philosophy is, or has ever pretended to be, a science. We advocate for an interdisciplinary investigation of consciousness that takes eidetic structures as the seeds of empirical hypotheses into neural function (qualia, on the other hand, would likely not be very productive of hypotheses. After all, how much is there to say about redness?). This can open the cognitive neuroscience of consciousness up to vistas of richer views of consciousness.

## Conclusion

In sum, we propose a novel, relativistic theory of consciousness, one that accounts for both the functional and phenomenal features of consciousness, bridging the explanatory gap. Through conceptual arguments and mathematical formalizations, we propose that there is no need to expand the basic inventory of nature (as dualists like Chalmers, 2017 argue), nor is there a need to explain away phenomenal features (as illusionists argue). Phenomenal features are not truly private, since the principle of relativity allows us to perform a transformation from one cognitive frame of reference to another. We provided a mathematic transformation between two idealized cognitive systems taken from different cognitive frames of reference, showing their relativistic equivalence. The privacy of phenomenal features is only an illusion, based on our biological limitations and the technological limitations of current science—basically, we can’t yet actually perform such a transformation. But our formalization is a proof of concept, showing that it is theoretically feasible. Since phenomenal features are not private, both the presence of zombies and the paradox of phenomenal judgment fall away. The dualist infers from these that phenomenal consciousness is a non-material extra force or property of nature, while the illusionist infers that phenomenal consciousness is merely an illusion created by phenomenal judgments. But once the privacy issue, zombies, and this paradox are neutralized, there is no longer any strong motivation for the dualist and illusionist positions. Phenomenal consciousness is neither private nor delusional, just relativistic.

Not only does the relativistic theory of consciousness legitimize the study of phenomenal features in science, but it furthermore opens up many new questions and possibilities for research. We noted that philosophers studying phenomenal consciousness could play a legitimate role in the science of consciousness, such as by theoretical contributions to experiments seeking the neural basis of phenomenal and eidetic structures.

## Data Availability Statement

The original contributions presented in the study are included in the article/Supplementary Material, further inquiries can be directed to the corresponding author.

## Author Contributions

Both authors listed have made a substantial, direct, and intellectual contribution to the work, and approved it for publication.

## Conflict of Interest

The authors declare that the research was conducted in the absence of any commercial or financial relationships that could be construed as a potential conflict of interest.

## Publisher’s Note

All claims expressed in this article are solely those of the authors and do not necessarily represent those of their affiliated organizations, or those of the publisher, the editors and the reviewers. Any product that may be evaluated in this article, or claim that may be made by its manufacturer, is not guaranteed or endorsed by the publisher.

## Footnotes

1 The terms are nearly synonymous, but ‘transcendental’ means additionally that one has performed the *epochç*. The *epochç* refers to a theoretical bracketing of the ontological status of phenomena—i.e., bracketing the existence of the world. The goal of the *epochç* is to not confuse external reality with the immanent features of phenomenal consciousness. Since this is not a concern of ours here, we stick with the term ‘eidetic.’ See Husserl (1925/2012).
