# Supplementary material for: A Relativistic Theory of Consciousness
Source: Front Psychol. 2022 May 12;12:704270. doi: 10.3389/fpsyg.2021.704270 (PMC9255957; doi:10.3389/fpsyg.2021.704270)
Supplement: Supplementary file 1 [file Data_Sheet_1.pdf]

A Relativistic Theory of Consciousness

Supplementary Material

Nir Lahav, Zachariah A. Neemeh

1 Supplementary Notes

1.1 Supplementary Note 1

In the paper we proved that (eq. 15) –

1. $\vec{l}^{\mu}_{p_{l,x_t}} = m^{\mu}$

Where m^{μ} is a result of a specific measurement of ALICE and $\vec{l}^{\mu}_{p_{l,x_t}}$ is a specific linguistic response of ALICE. Now we can plug this result back in equation 13 from the paper:

2. $\vec{l}^{\nu}_{p_{l,x_t}} = \widetilde{\Lambda}^{\nu\mu} m^{\mu}$,

and get –

3. $\vec{l}^{\nu}_{p_{l,x_t}} = \widetilde{\Lambda}^{\nu\mu}\, \vec{l}^{\mu}_{p_{l,x_t}}$

which gives us a similar equation to equation 12 from the paper, but to a specific linguistic response. If we will repeat this process to every linguistic response of the two systems we will get:

4. $\cup\, \vec{l}^{\nu}_{p_{l,x_t}} = \widetilde{\Lambda}^{\nu\mu} \cup \vec{l}^{\mu}_{p_{l,x_t}}$,

Where U is the unification operator over all specific linguistic responds.

Same process can be done for the case of ALICE's qualia.

1.2 Supplementary Note 2

We want to expend equation 34 from the paper (here it will be equation 1):

1. $\vec{c}^{\mu}_{p_{l,c_{x_t}}} = Q^{\mu}_{Q_l,Q_{x_t}}$,

For different kinds of qualia (here it's a second order quale. A quale about the previous quale – experience of an experience). Let's choose another equation o describe the first-person perspective. For example, from the first-person perspective, quale can cause the next thought:

$$2. {Q^{\nu}}_{Q_l,Q_{x_t}} = \bar{F}\left(Q_l, Q_{x_t}\right)^{\nu} .$$

From Alice's first-person perspective, she feels happy (quale Q_{x_t}) and she experience a question, $Q_l =$ "What are you experiencing right now?" as a result she experiences herself trying to answer the question in her head (a second order quale, ${Q^{\nu}}_{Q_l,Q_{x_t}}$ = I feel happiness). The general function $\bar{\mathrm{F}}$ maps the two qualia to the quale of the answer ${Q^{\nu}}_{Q_l,Q_{x_t}}$. Notice that in contrary to the example in the paper, this case is all in Alice's mind with no linguistic response.

Because of the equivalence between Alice and ALICE we can change the cognitive frame of reference in equation 1 from the frame of ALICE (μ) to the frame of Alice (ν) using a delta function:

$$3. \ \delta^{\nu\mu}\vec{c}^{\mu}_{p_l,c_{x_t}} = \delta^{\nu\mu}Q^{\mu}_{Q_l,Q_{x_t}} ,$$

$$4. \ \vec{c}^{\nu}_{p_l,c_{x_t}} = Q^{\nu}_{Q_l,Q_{x_t}} .$$

Now we see that like in the frame of ALICE, there is an identity between a quale and the corresponding phenomenal judgement representation also in the frame of Alice. Now, we can plug this result (4) to the equation of the creation of the next quale (2):

$$5. \ \vec{c}^{\nu}_{p_l,c_{x_t}} = \bar{F}\left(Q_l, Q_{x_t}\right)^{\nu} .$$

From equation 22 in the paper we know that:

$$6. \ \vec{c}^{\nu}_{p_l,c_{x_t}} = C\left(\vec{p}_l, \vec{c}_{x_t}, E, W, M, A, I\right)^{\nu}$$

Where $\vec{p}_l$ is the sentence comprehension representation of the question "What are you experiencing right now?" created by the linguistic module, $\vec{c}_{x_t}$ is the phenomenal judgment of being happy and $\vec{c}^{\nu}_{p_l,c_{x_t}}$ is the phenomenal judgment of the answer to the question (I feel happiness). However, if we want to be more accurate we need to split this equation into two equations. First, Alice has a process of creating a phenomenal judgment of the question:

$$7. \ \vec{c}^{\nu}_{l} = C(\vec{p}_l, E, W, M, A, I)^{\nu}$$

Where $\vec{c}^{\nu}_{l}$ is the phenomenal judgment of the question "What are you experiencing right now?". Only then, after she has phenomenal judgment about the question, she creates an answer:

$$8. \ \vec{c}^{\nu}_{c_l,c_{x_t}} = C\left(\vec{c}_l, \vec{c}_{x_t}, E, W, M, A, I\right)^{\nu} .$$

Where $\vec{c}^{\nu}_{c_l,c_{x_t}}$ is the phenomenal judgment of the answer (I feel happiness) to the question $\vec{c}^{\nu}_{l}$ ("What are you experiencing right now?") according to $\vec{c}_{x_t}$ (feeling of happiness).

We can use equation 8 and write equation 5 again:

7. $C\left(\vec{c}_l, \vec{c}_{x_t}, E, W, M, A, I\right)^{\nu} = \bar{F}\left(Q_l, Q_{x_t}\right)^{\nu}$.

Once again, we can identify $\mathrm{C} = \bar{\mathrm{F}}$, and

8. $\vec{c}_{\mathrm{l}}^{\nu} = Q_{\mathrm{l}}^{\nu}$ and $\vec{c}_{x_t}^{\nu} = Q_{x_t}^{\nu}$

Here we expended equation 1 by proving that in frame ν, a quale about linguistic response, Q_{l}^{ν} is identical to the phenomenal judgement representation of the same linguistic response, $\vec{c}_{\mathrm{l}}^{\nu}$ and that a quale about arbitrary input, $Q_{x_t}^{\nu}$ is identical to the phenomenal judgement representation of the same arbitrary input, $\vec{c}_{x_t}^{\nu}$. Hence, we generalized equation 1 to all possible qualia.

1.3 Supplementary Note 3

A similar scenario that demonstrates the importance of the spatial positions condition can be made by choosing the identity transformation for a cognitive system that measures a replica of its own quale by the sensation subsystem. Without losing generality, let's assume that Bob sees in front of him a copy of the appropriate firing patterns of his brain while thinking of an apple (a copy of his state $\vec{x}_t = \sum_{m=1}^{j} \alpha_m \hat{e}_m$. For example, Bob sees the exact firing patterns of his brain while thinking of an apple). As a result, he will measure the physical properties of his quale, $\vec{Q}_{x_t}^{\nu} = \vec{Q}_{\sum \alpha_m \hat{e}_m}^{\nu}$ (equation 41 in the paper). When we apply the transformation function as identity transformation it should not change anything and give back the same quale of the physical properties, $\vec{Q}_{x_t}^{\nu} = \vec{Q}_{\sum \alpha_m \hat{e}_m}^{\nu}$ (equation 47 in the paper). Indeed, the transformation function checks if the physical properties of the quale are a state in the state space of C^{ν}. Because the input is a copy of the physical properties of the quale of this frame, the answer is positive and the matching function will return something different then the empty set, ${m_C}^{\nu}(\vec{x}_t) \neq \emptyset$. But because the system observes its quale by the sensation subsystem it means that this input is outside of the cognitive system (the sensation subsystem does not sense inputs from within the brain). Consequently, $\delta^{\vec{r}_0{}^{\nu}\vec{r}_{x_t}{}^{\nu}} = 0$ and the transformation function will send $\vec{x}_t$ to the sensation subsystem until eventually the appropriate quale $\vec{Q}_{x_t}^{\nu} = \vec{Q}_{\sum \alpha_m \hat{e}_m}^{\nu}$ will be made as desired.

1.4 Supplementary Note 4

The inverse transformation function, from frame of reference ν to frame μ, is:

1. $\vec{Q}_{x_t}^{\mu} = \Lambda^{\mu\nu}\left(\vec{Q}_{x_t}^{\nu}\right)$

2. $\Lambda^{\mu\nu}(\vec{x}_t, S^{\mu}) = \begin{cases} {m_C}^{\mu}(\vec{x}_t) = \emptyset \vee \delta^{\vec{r}_0{}^{\mu}\vec{r}_{x_t}{}^{\mu}} = 0\,, S^{\mu}(\vec{x}_t) \\ {m_C}^{\mu}(\vec{x}_t) \neq \emptyset \wedge \delta^{\vec{r}_0{}^{\mu}\vec{r}_{x_t}{}^{\mu}} = 1\,, C^{\mu}(\vec{x}_t) \end{cases}$

It is easy to show that the transformation function and the inverse transformation function cancel each other out, as expected, when we apply a function composition:

3. $\Lambda^{\nu\mu}\left(\Lambda^{\mu\nu}(\vec{Q}^{\nu}_{x_t})\right) = \vec{Q}^{\nu}_{x_t} \Leftrightarrow \Lambda^{\mu\nu} = (\Lambda^{\nu\mu})^{-1}$

$\Lambda^{\mu\mu}$ (and also $\Lambda^{\nu\nu}$) is the inverse of itself. If we want to write it for the special scenario of ALICE and Alice where the difference between the spatial positions of the cognitive systems, r_0, r'_0 breaks the symmetry of $\Lambda^{\mu\mu}$, we can add the positions of the systems as part of the transformation function. For example, for frame ν:

5. $\Lambda^{\nu_{r_0}\nu_{r'_0}} = (\Lambda^{\nu_{r'_0}\nu_{r_0}})^{-1}$
